# Elevated Alpha-Synuclein Impairs Innate Immune Cell Function and Provides a Potential Peripheral Biomarker for Parkinson's Disease

**DOI:** 10.1371/journal.pone.0071634

**Published:** 2013-08-23

**Authors:** Shyra J. Gardai, Wenxian Mao, Birgitt Schüle, Michael Babcock, Sue Schoebel, Carlos Lorenzana, Jeff Alexander, Sam Kim, Heather Glick, Kathryn Hilton, J. Kent Fitzgerald, Manuel Buttini, San-San Chiou, Lisa McConlogue, John P. Anderson, Dale B. Schenk, Frederique Bard, J. William Langston, Ted Yednock, Jennifer A. Johnston

**Affiliations:** 1 Elan Pharmaceuticals, Research, South San Francisco, California, United States of America; 2 The Parkinson's Institute, Sunnyvale, California, United States of America; Foundation for Biomedical Research Academy of Athens, Greece

## Abstract

Alpha-synuclein protein is strongly implicated in the pathogenesis Parkinson's disease. Increased expression of α-synuclein due to genetic multiplication or point mutations leads to early onset disease. While α-synuclein is known to modulate membrane vesicle dynamics, it is not clear if this activity is involved in the pathogenic process or if measurable physiological effects of α-synuclein over-expression or mutation exist *in vivo*. Macrophages and microglia isolated from BAC α-synuclein transgenic mice, which overexpress α-synuclein under regulation of its own promoter, express α-synuclein and exhibit impaired cytokine release and phagocytosis. These processes were affected *in vivo* as well, both in peritoneal macrophages and microglia in the CNS. Extending these findings to humans, we found similar results with monocytes and fibroblasts isolated from idiopathic or familial Parkinson's disease patients compared to age-matched controls. In summary, this paper provides 1) a new animal model to measure α-synuclein dysfunction; 2) a cellular system to measure synchronized mobilization of α-synuclein and its functional interactions; 3) observations regarding a potential role for innate immune cell function in the development and progression of Parkinson's disease and other human synucleinopathies; 4) putative peripheral biomarkers to study and track these processes in human subjects. While altered neuronal function is a primary issue in PD, the widespread consequence of abnormal α-synuclein expression in other cell types, including immune cells, could play an important role in the neurodegenerative progression of PD and other synucleinopathies. Moreover, increased α-synuclein and altered phagocytosis may provide a useful biomarker for human PD.

## Introduction

The evidence implicating alpha-synuclein (α-syn) in the pathogenesis of Parkinson's Disease (PD) is overwhelming. However, there is not a clear consensus on the manner in which α-syn leads to pathology in PD and other synucleinopathies. Alpha-synuclein is a major component of Lewy bodies (LBs), and descriptions of α-syn overexpression leading to aggregation are abundant [1]–[3]. Human genetic data have demonstrated that missense mutations and multiplications in the α-syn gene cause familial PD [4], [5]. In the case of gene multiplication, increased levels of α-syn protein are presumed to result in a dominant gain-of-function that leads to pathology. While increased levels of α-syn may lead to aggregation and toxicity, research over the past few years has also revealed that elevated α-syn can interfere with the creation, localization, and/or maintenance of vesicle pools [6]–[9].

Although α-syn dysfunction is largely studied in neurons, α-syn pathology in PD patients has been described in non-CNS cell types and organ systems [10], [11] and the current perspective on PD is evolving from a purely dopaminergic neuron brain disease to a more systemic disease [12], [13]. Altered microglia responses are a hallmark of PD pathology and recent evidence suggests that reactive microglia represent more than a response to neuronal injury, and may actively participate in the demise of dopaminergic neurons [14]. Given a clear role for α-syn in PD pathology, a number of studies have examined the response of microglia to extracellular α-syn, but few studies have explored the function and potential pathogenic role of α-syn expressed by microglial cells themselves [15], [16]. Interestingly, mice in which the α-syn gene was knocked out were reported to have changes in microglial function, specifically, altered phagocytosis and increased cytokine response [17].

Engulfment and removal of pathogens and cellular debris is the quintessential function of phagocytes. Phagocytosis is a key process executed by both professional phagocytes (macrophages and microglia) as well as non-professional phagocytes (such as fibroblasts and endothelial cells). Phagocytosis is required for the efficient elimination and destruction of pathogens, timely removal of dead and dying cells, cellular sampling of environmental antigens for presentation to adaptive immune cells, and maintenance of tissue homeostasis [18], [19]. This process *in vivo* is so efficient, that the presence of dead, dying, and apoptosing cells is generally absent, and can only be observed under conditions of impaired cellular clearance by phagocytes.

Engulfment of medium and large sized particles involves internalization of large expanses of plasma membrane, coupled with addition of new plasma membrane to maintain the surface area and shape of the cell [20]. Elegant experiments utilizing membrane labeling compounds or electrophysiological methods demonstrated that the maintenance of surface area during particle ingestion is linked to the concomitant mobilization and focal exocytosis of membrane to the cell surface [21], [22]. While the intracellular source of the replacement membrane varies between different systems, they all share a common process in which the delivery of additional membrane through vesicular fusion is coordinated by various members of the SNARE family [23]. SNARE family members associate into stable complexes that direct membrane fusion events. The SNARE mediated process of vesicle exocytosis is not only critical for phagocytosis but is also required for the release of inflammatory cytokines from macrophages and microglia, [24].

SNARE complexes prime vesicles for fusion with the plasma membrane and are composed of proteins from each of three separate families: a SNAP, a Syntaxin, and a VAMP. SNARE complexes are formed by cells in anticipation of membrane fusion events and upon stimulation, the complexes localize to the points of vesicle contact, facilitate membrane fusion, and then dissociate into their individual components that are recycled to form new complexes. Numerous studies in other labs have shown that α-syn may affect SNARE complex assembly either through direct interaction with members of the SNARE family, or by sequestering agents, such as arachadonic acid, which promote SNARE assembly and activity [25], [26].

We have recently developed a novel transgenic genomic mouse model in which wild type or the E46K mutant form of human α-syn was expressed from a human bacterial artificial chromosome (BAC). This model is similar to that created by Kou et al. [27] however, we focused on wild type and the E46K mutated form of α-syn rather than the A30P and A53T mutations described previously. The BAC construct contains the α-syn promoter and other potential regulatory regions including upstream, and 15 kb downstream sequences. Because α-syn expression is driven from its own promoter, not from a highly active exogenous promoter, this *in vivo* system may provide insight into human disease, because in humans duplication or triplication of the gene is sufficient to lead to early onset PD. In addition, the potential impact of elevated α-syn levels on multiple cellular and tissue systems can be assessed. In the work described here, we used these mice to examine the role of α-syn in innate immune cell function. We were particularly interested in macrophages and microglia because of their involvement in PD pathology and their intensive reliance on membrane dynamics for their central roles in cytokine release and phagocytic function [23], [28].

Our data demonstrates that increased expression of the α-syn protein has an inhibitory effect on the release of inflammatory cytokines, vesicular fusion, and phagocytic processes. Defective phagocytosis occurred in brain microglia and peripheral macrophages, either isolated and assayed in culture, or assayed *in situ*. Fibroblasts and monocytes isolated from sporadic PD patients and a patient carrying a triplication of the α-syn gene exhibited elevated α-syn levels, which correlated with defective phagocytic function. We also expand upon the molecular details regarding α-syn interaction with membrane trafficking components, and demonstrate active recruitment of α-syn to a site of stimulated membrane fusion during phagocytosis. Our data further defines the role that α-syn plays in membrane vesicle dynamics and extends these findings into systemic processes beyond neuronal function, which may provide a valuable peripheral biomarker of elevated α-syn levels and dysfunctional vesicle dynamics. Our data suggest that there may be a previously unappreciated involvement of innate immune cell function in synucleinopathies, and that cells of the innate immune system provide an excellent, tightly regulated cellular model in which to study the effects of increased α-syn levels on vesicle function.

## Results

### α-syn is expressed in microglia from BAC transgenic mice

**Figure 1:**
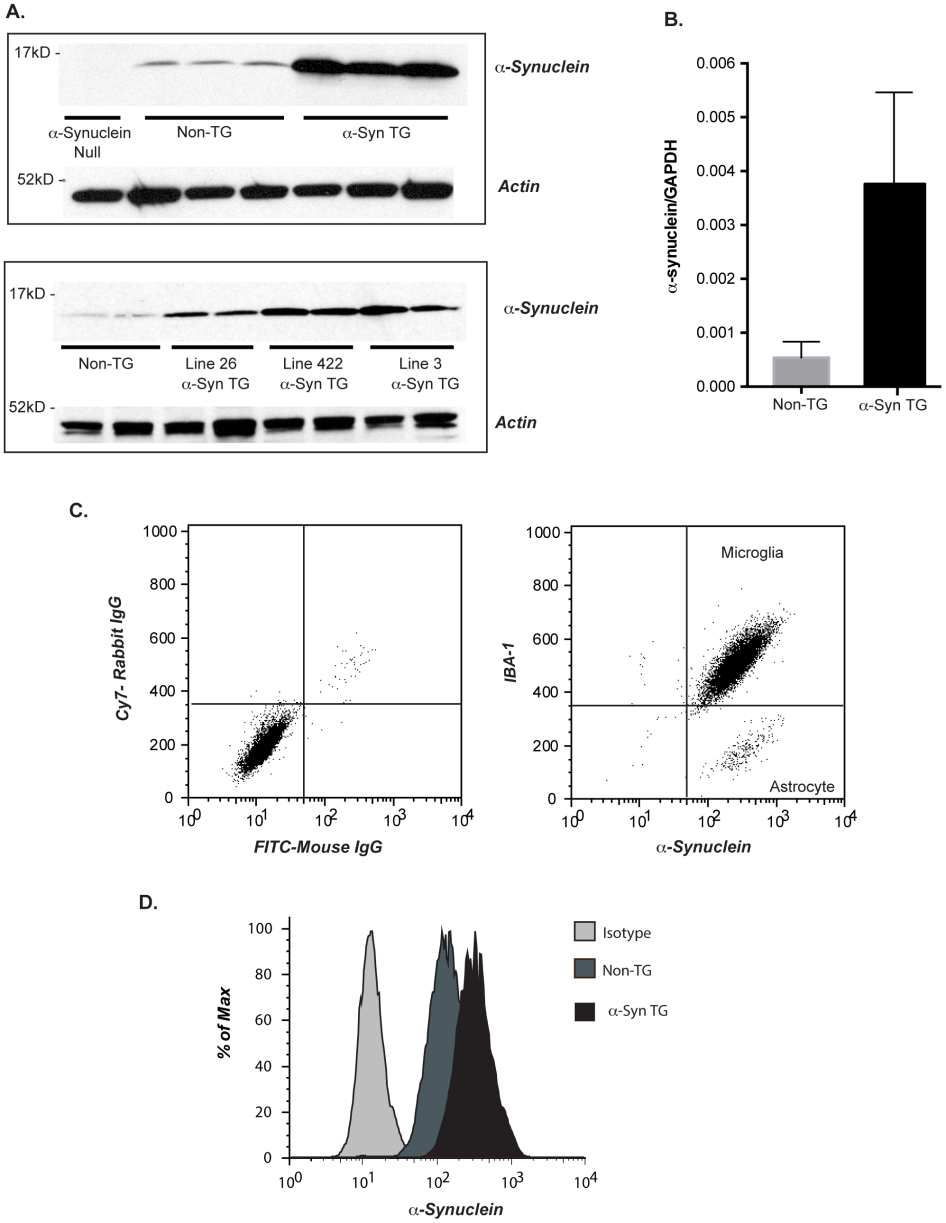
Alpha-synuclein levels are increased in BAC transgenic microglia. (**A**) **Top panel:** Microglia containing a BAC construct of human α-syn (line 26) have elevated levels of α-syn protein compared to littermate controls. Expression is specific as shown by absence of detectable α-syn in lysates from α-syn null cell, and loading is equivalent as evidenced by equal actin levels (gel is representative of 3 independent experiments). **Bottom panel:** Microglia isolated from three independently-derived α-syn BAC lines (line 422;26; and 3) have elevated α-syn expression compared to non-TG littermates and equivalent loading is demonstrated by equal actin (gel is representative of 3 independent expts). (**B**) Alpha-synuclein mRNA was assessed by rt-PCR in line 26 non-TG and α-syn TG microglia. Levels were normalized to GAPDH and the ratio for Line 26 α-syn TG and non-TG are shown here (n = 10 pups/GT/expt. +/− s.e.m *P≤0.001 when α-syn TG samples were compared with non-TG. (**C**) FACS analysis of line 26 microglial cultures stained with IBA1 as a specific marker for microglia, and 5C12 for intracellular α-syn. Compared to isotype controls microglia express α-syn, and are the predominant cell type accounting for α-syn expression in the cultures. (**D**) Microglia isolated from line 26 and non-TG littermate were stained for α-syn and levels assessed by flow cytometry. An isotype-matched antibody on α-syn TG cells was used as a negative control (histogram is representative of 3 independent expts).

To examine the impact of α-syn overexpression on innate immune cell function, we utilized three transgenic mouse lines, each of which expresses α-syn from a bacterial artificial chromosome (BAC) genomic context; line 422 expressing wild type α-syn and two lines, line 26 and line 3, expressing the mutant E46K form. The BAC transgene results in an increase in α-syn expression (as measured by RT-PCR and ELISA) and a full characterization of these animals will be reported elsewhere (Hilton et al., in preparation). Because the expression of α-syn is driven by endogenous regulatory sequences, we first examined if human α-syn was detectable in mouse microglia isolated from genomic mice, and whether expression was increased compared to wild type microglia. Non-transgenic (non-TG) and α-syn genomic transgenic (α-syn TG) microglia expressed α-syn detectable by immunoblot and the specificity was validated with microglia isolated from α-syn null mice (**Figure 1A** **, top panel**). Microglia isolated from all three α-syn TG lines (3, 26, and 422) expressed elevated levels of human α-syn as confirmed by mRNA analysis (representative line26 samples shown here Figure 1B) and total α-syn protein as shown by immunoblot compared to non-TG littermates **(** **Figure 1A** **, bottom panel)**. Since α-syn expression can occur in astrocytes [29] we verified microglial cell expression by flow cytometry, double labeling cells with anti-α-syn and a microglia marker, IBA-1 (**Figure 1C**). Specific microglia α-syn expression as determined by intracellular staining was increased in cells from α-syn TG animals (**Figure 1D**). These results also show that microglial cells are the predominant cell type (and hence the predominant source of α-syn) in our cultures.

### Increased α-syn in microglia and macrophages disrupts cytokine exocytosis

**Figure 2:**
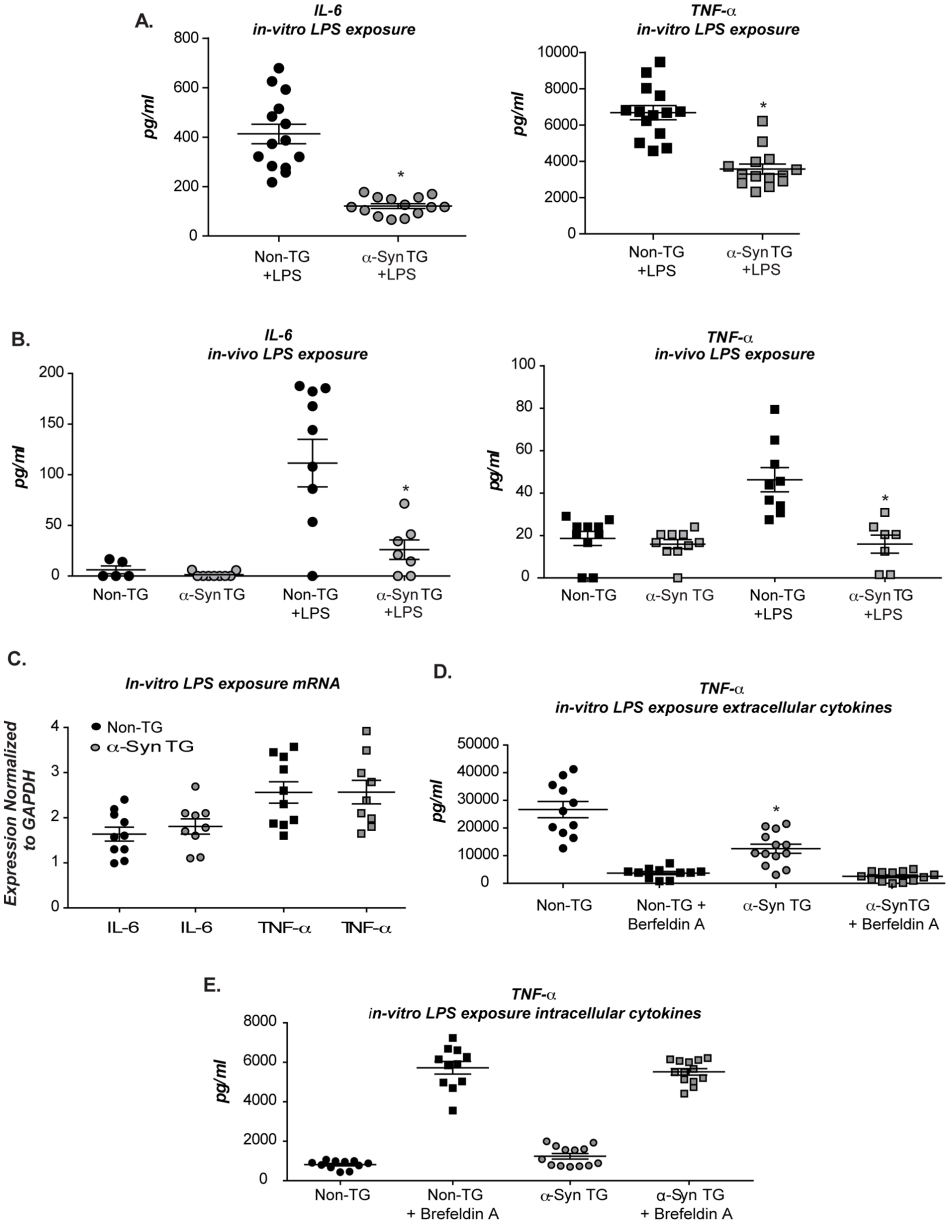
Alteration in cytokine secretion in BAC transgenic mice. In all graphs, each dot represents measurements from cells isolated from an individual pup or animal (**A**) Microglia from line 26 and non TG littermates were stimulated with LPS and TNFα and IL6 measured by ELISA (n = 2; 7 pups/GT/expt +/− s.e.m *P≤0.005 when α-syn TG samples were compared with non-TG). (**B**) Microglia isolated from three independent human α-syn BAC TG mouse lines, (line 422; 26; and 3) and their corresponding non-TG littermate controls. Cells were stimulated as above and measurements were made for TNFα by ELISA (n = 2; 3 pups/GT/expt +/− s.e.m *P≤0.05 when α-syn TG samples were compared with non-TG). (**C**) Line 26 TG mice and their non-TG littermates received an injection of low dose LPS for 6 months and serum inflammatory cytokines (TNFα and IL6) were measured by luminex multiplex analysis (n = 1; 7–10 mice/GT/expt +/− s.e.m* P≤0.05 when α-syn TG samples were compared with non-TG). (**D**) Microglia from line 26/syn ^null^ or α-syn ^null^ littermates mice were stimulated with LPS and cytokine expression for IL6 and TNFα assessed at the mRNA level by multiplex analysis (n = 2; 5 pups/GT/expt +/− s.e.m *p≤0.001 when α-syn TG samples were compared with non-TG). (**E+F**) Microglia isolated from line 26/syn ^null^ or α-syn ^null^ littermates were stimulated with LPS in the presence or absence of Brefeldin A. Tissue culture supernatant (**E**) or cell lysate (**F**) was assessed for TNFα production by ELISA (n = 2; 5 pups/GT/expt +/− s.e.m *p≤0.001 when α-syn TG samples were compared with non-TG).

Microglia deficient in α-syn were reported to have altered cytokine release [17] prompting us to investigate the consequence of elevated expression on cytokine secretion. To induce cytokine release, microglia from line 26 TG and non-TG littermate controls were exposed to LPS for 18hrs. Microglia from the α-syn TG mice secreted significantly less IL-6 and TNF-α compared to their non-TG controls (**Figure** **2A**), which is in contrast with increased cytokine secretion reported from α-syn null cells [17]. These deficits were not due to aberrant insertion of the BAC transgene into a cytokine locus, or other interference, since the same effects on cytokine release were measured in microglia isolated from all three independent BAC transgenic lines (lines 3, 26 and 422) (**Figure S1**). The deficit was also not due solely to the E46K mutation of α-syn, since line 422 expresses the WT α-syn sequence. Alterations in cytokine secretion were also found *in vivo*, where α-syn TG mice had muted levels of circulating inflammatory cytokines following low dose lipopolysaccharides (LPS) administration (**Figure 2B**).

Secretion of specific cytokines requires dynamic vesicle mobilization and fusion of cargo containing vesicles with the plasma membrane. Therefore, we investigated whether α-syn altered innate immune responsiveness by negatively regulating vesicle dynamics, or by affecting signaling pathways upstream of membrane events, such as LPS induced toll-like receptor (TLR) signaling. To distinguish between these two possibilities, cytokine mRNA production in non-TG and α-syn TG microglia was evaluated following LPS exposure. Microglia from human α-syn TG animals crossed onto a murineα-syn null background were utilized to limit potential confounding effects of endogenous murine α-syn *(line 26 x α-syn null)*. LPS stimulated equivalent cytokine mRNA production in non-TG and α-syn TG microglia (**Figure 2C**). Furthermore, we confirmed that the non-TG and α-syn TG cells made similar levels of cytokines at the protein level by blocking cytokine release with Brefeldin A (BFA) and measuring intracellular cytokine levels (**Figure 2D** **and** **Figure 2E**). These data confirm that equivalent levels of cytokines are produced in response to LPS, and presumably loaded into vesicles, but are released at reduced levels from cells with α-syn overexpression.

### Increased α-syn impairs phagocytic processes

**Figure 3:**
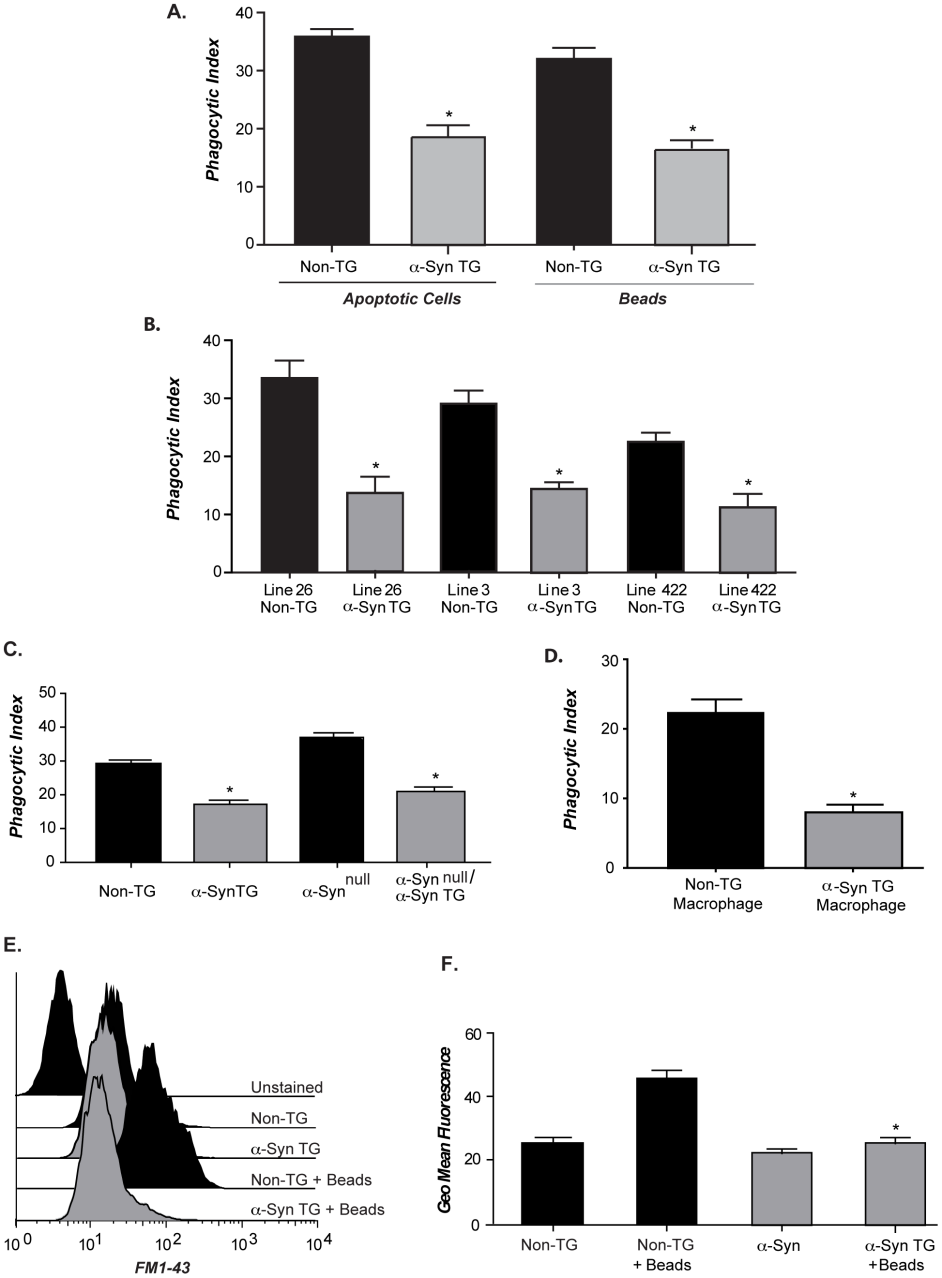
Increased levels of α-syn affect microglial phagocytosis. (**A**) Microglia from line 26 TG or non-TG littermates were incubated with 10 µ beads or apoptotic Jurkat T-cells for 90 minutes. A phagocytic index was calculated by microscopic visualization (n = 3; 3 pups/GT/expt +/− s.e.m *p≤0.001 when α-syn TG samples were compared with non-TG). (**B**) Microglia from line 26/syn ^null^ or α-syn ^null^ littermates were incubated with 10µ beads and a phagocytic index calculated (n = 3; 3 pups/GT/expt +/− s.e.m *p≤0.002 when α-syn TG samples were compared with non-TG). (**C**) Peritoneal macrophages isolated from line 26 TG or non-TG littermates were cultured with apoptotic Jurkat T-cells and a phagocytic index was calculated (n = 2; 5 pups/GT/expt +/− s.e.m *p≤0.001 when α-syn TG samples were compared with non-TG). (**D**) Microglia isolated from line 26 TG or non-TG littermates were left unfed or fed beads followed by FM1-43 addition on ice for 10 minutes, fluorescence was assessed by flow cytometry under resting and stimulated (plus bead) conditions, (histogram is representative of 3 independent expts). (**E**) Geometric mean fluorescence of FM1-43 incorporation in resting and stimulated cell was calculated (n = 3; 3 pups/GT/expt +/− s.e.m *p≤0.001 when FM1-43 incorporation between wild type and line 3 microglia stimulated with beads was compared).

Elegant experiments demonstrated that phagocytosis of large particles requires the mobilization and exocytosis of membrane at the cell surface at a magnitude similar to or greater than that required for cytokine release [22]. Therefore, microglia from human α-syn TG mice were isolated and assayed for phagocytic capacity as described in Materials and Methods. Compared to non-TG littermates, microglia overexpressing human α-syn exhibited significantly impaired phagocytosis of both beads and apoptotic cells (**Figure 3A**).

Phagocytosis of apoptotic cells requires engagement of specific receptors, while ingestion of inert particles such as latex beads is believed to be independent of receptor expression and engagement. Because ingestion of both beads and cells was decreased in the human α-syn TG cells, it is unlikely to be caused by a specific receptor defect and more likely caused by a failure in membrane mobilization and function. The observed phagocytic deficit was also likely not due to insertion artifacts of the BAC construct since the three α-syn TG lines examined exhibited a similar reduction in phagocytosis (**Figure 3B**), and occurred with both wild type α-syn (line 422) and the E46K α-syn mutation (lines 3 and 26). Phenotypes associated with human α-syn overexpression may be exacerbated in the absence of murine α-syn. However, the absence of murine α-syn did not amplify the phagocytic defect associated with human α-syn overexpression (**Figure 3C**) nor did it affect the deficit in cytokine secretion (Figure 2E). The phagocytic defect was also not restricted to microglia or to cells isolated during early development as peritoneal macrophages isolated from adult human α-syn TG animals also exhibited decreased phagocytosis (**Figure 3D**). Collectively, these data suggest that α-syn impairs large particle phagocytosis in microglia and macrophages.

Membrane addition to the cell surface in the initial phase of phagocytosis can be tracked with FM1-43, a fluorescent dye that incorporates into exposed membranes and hence provides increased fluorescence during the processes of phagocytosis. With non-TG microglia, FM1-43 incorporation and fluorescence increased after bead addition, however, FM1-43 membrane incorporation and fluorescence did not increase in α-syn TG microglia after bead addition of beads (**Figure 3E**). Alterations in FM1-43 labeling were quantified as mean fluorescence intensity compared to unlabeled cells from 3 independent experiments (**Figure 3F**) and this data suggest that increased α-syn results in defects in vesicle recruitment to the plasma membrane.

### Elevated α-syn results in defective cell clearance *in vivo*

**Figure 4:**
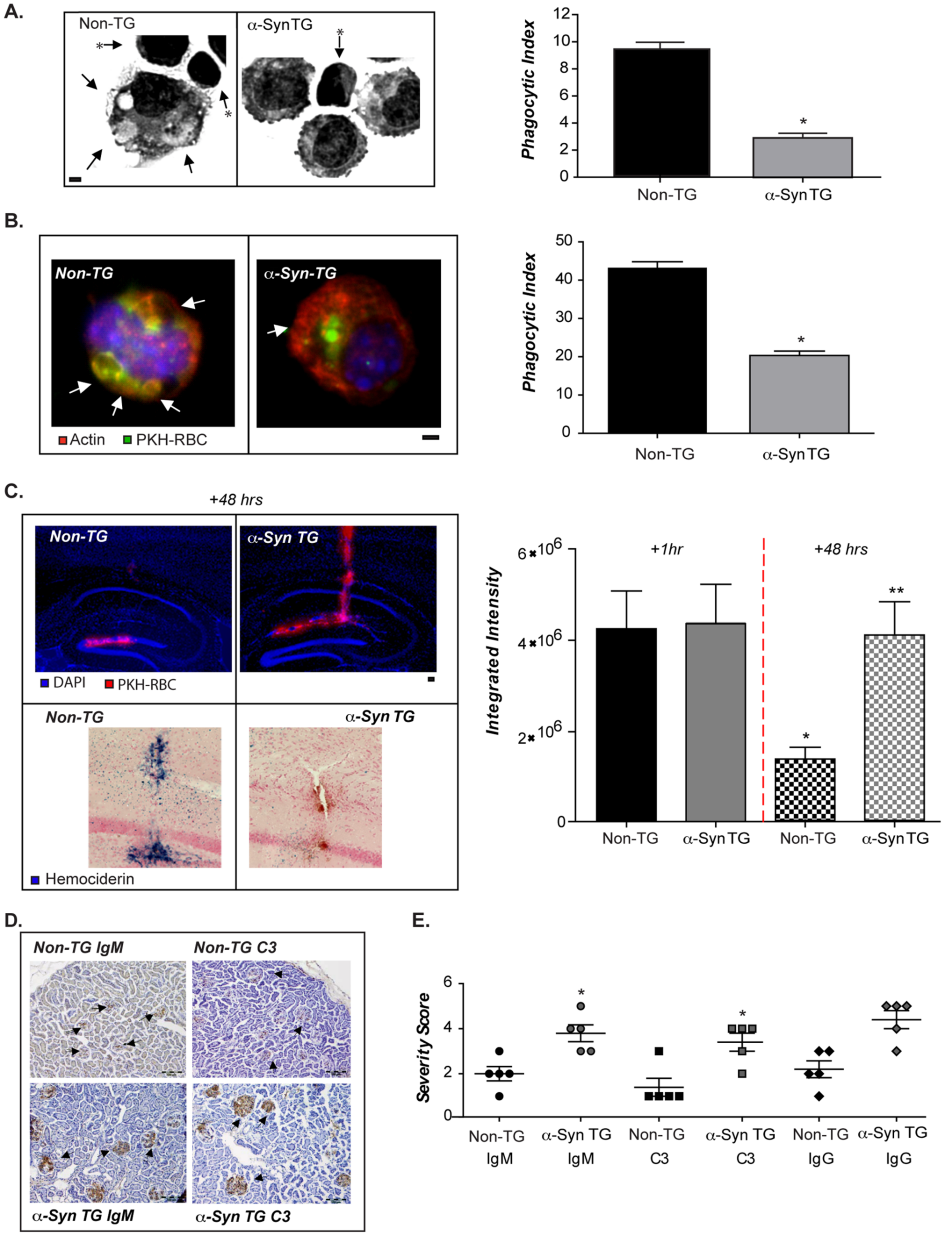
Altered phagocytosis in-vivo in α-syn BAC transgenic animals. (**A**) Representative images (left) and quantified phagocytic index (right) of peritoneal macrophages exposed in vivo to apoptotic Jurkat T-cells in line 26 and non-TG littermate mice (n = 2; 5 mice/GT/expt +/− s.e.m *p≤0.001 when α-syn TG samples were compared with non-TG). Scale bars are equal to 1 micron. Arrows point towards ingested apoptotic cells and asterisked arrows towards bound/non-ingested cells. (**B**) Representative images (left) and quantified phagocytic index (right) of peritoneal macrophages exposed in vivo to fluorescent CD47−/− RBC's (red) in line 3 and non-TG littermates (n = 2; 5 mice/GT/expt+/− s.e.m *p≤0.001 when α-syn TG samples were compared with non-TG). Scale bars are equal to 1 micron. Arrows point towards ingested fluorescent RBC, which appear yellow as they are digested within the green labeled cells (FITC-phalloidin). (**C**) Stereotactic injection of red fluorescent RBC's into the hippocampus of line 3 and non-TG littermate mice. Fluorescence was visualized (upper image) and quantified (bar graph). Particle removal is represented as reduced fluorescence. RBC uptake and degradation was followed as the appearance of hemosiderin (lower image), (n = 3; 3 mice/GT/expt +/− s.e.m * p≤.0.001 compared to 1 hr non-TG ** compared to non-TG at 48hrs). Scale bars are equal to 10 microns. (**D**) Kidneys from line 26 and littermate non-TG female mice were stained for C3 and IgM. (**E**) Intensity of antibody was quantified by a certified pathologist in non-TG and α-syn TG kidneys (n = 5 animals/GT +/−s.e.m *p<0.01 when α-syn TG samples were compared with non-TG).

*In vivo*, apoptotic cells are rapidly removed; however, with deficient clearance, apoptotic cells persist and undergo secondary necrosis, releasing potentially toxic and antigenic intracellular content [18], [19]. Since the data above (Figure 3) suggested that elevated expression of human α-syn altered phagocytosis *in vitro*, we examined clearance of cellular debris *in vivo*, comparing non-TG and α-syn TG mice. Using a well validated experimental system for macrophage clearance activity [30]–[32] animals were challenged with intraperitoneal injections of apoptotic Jurkat T-cells. Examination of peritoneal macrophages showed that *in vivo* clearance of apoptotic cells was significantly lower in mice overexpressing human α-syn (**Figure 4A**, micrographs and bar graph). Macrophages from non-TG mice contained highly compacted apoptotic cells engulfed within cytoplasmic phagosomes (**Figure 4A**, arrows), whereas non-engulfed apoptotic cells remained attached to the surface of macrophages in α-syn TG mice (**Figure 4A**, starred arrows). We also noted that non-TG macrophages appeared larger than α-syn TG cells, likely due to the increase in cell volume that accompanies phagocytosis [22]. The apparent difference in cell volume correlated with altered FM1-43 labeling in α-syn TG microglia as described above (**Figure 3E**).

The magnitude of the phagocytic deficit was quantified and expressed as a phagocytic index, revealing than macrophages in α-syn TG mice exhibited a 50% decrease in apoptotic cell uptake (**Figure 4A** graph, see Methods). An alternative model to assess *in vivo* phagocytosis in the peritoneum exploits CD47 deficient red blood cells (RBCs) [33] and using this model we observed a similar reduction in phagocytosis (**Figure 4B**). In this assay, the RBC were labeled with a red dye (PKH) and when engulfed appear yellow as the dye diffuses within the green labeled cells (FITC–phalloidin).

Due to altered phagocytosis in the periphery, we investigated whether microglia *in situ* also exhibited reduced particle engulfment. Fluorescent CD47^−/−^ RBCs were stereotactically injected into the cortex and hippocampus of non-TG and α-syn TG mice. Tissue was assessed for PKH fluorescence 1 or 48hrs after the injection (**Figure 4C** **,** upper panels). While equivalent levels of fluorescence were observed at 1hr following injection, 48hrs later, α-syn TG animals still exhibited significant PKH fluorescence, indicative of reduced clearance of the RBCs (**Figure 4C**). The loss of fluorescence in non-TG animals coincided with an increase in hemosiderin deposition, a known maker of RBC degradation (**Figure 4C**, lower panel).

A hallmark of defective apoptotic cells clearance *in vivo* is the production and deposition of anti-nuclear antibodies in the kidney, resulting in complement activation and glomerular nephritis, largely in female mice for reasons that are still unclear [34], [35]. Based on the defective apoptotic cell removal observed in α-syn TG mice we examined the serum of female α-syn TG mice for anti-nuclear antibodies and found elevated levels (Data not shown). Further, we examined kidneys of the BAC transgenic female mice and found that glomerular staining for complement C3 and IgM was increased compared to the non-TG controls (**Figure** **4D** **)**. Antibody and complement accumulation in the glomeruli of the α-syn TG animals was accompanied by mild mononuclear cell infiltration, suggesting that the phagocytic deficit caused by α-syn overexpression *in vivo*, has a pro-inflammatory impact. Quantitative analysis by a certified pathologist revealed increased deposition of antibodies and complement in the kidneys **(** **Figure 4E** **)** similar to what has been described for other disorders with defective apoptotic cell clearance [32].

### Alpha-synuclein levels drive reduced phagocytosis in macrophage and neuroglioma cells

**Figure 5:**
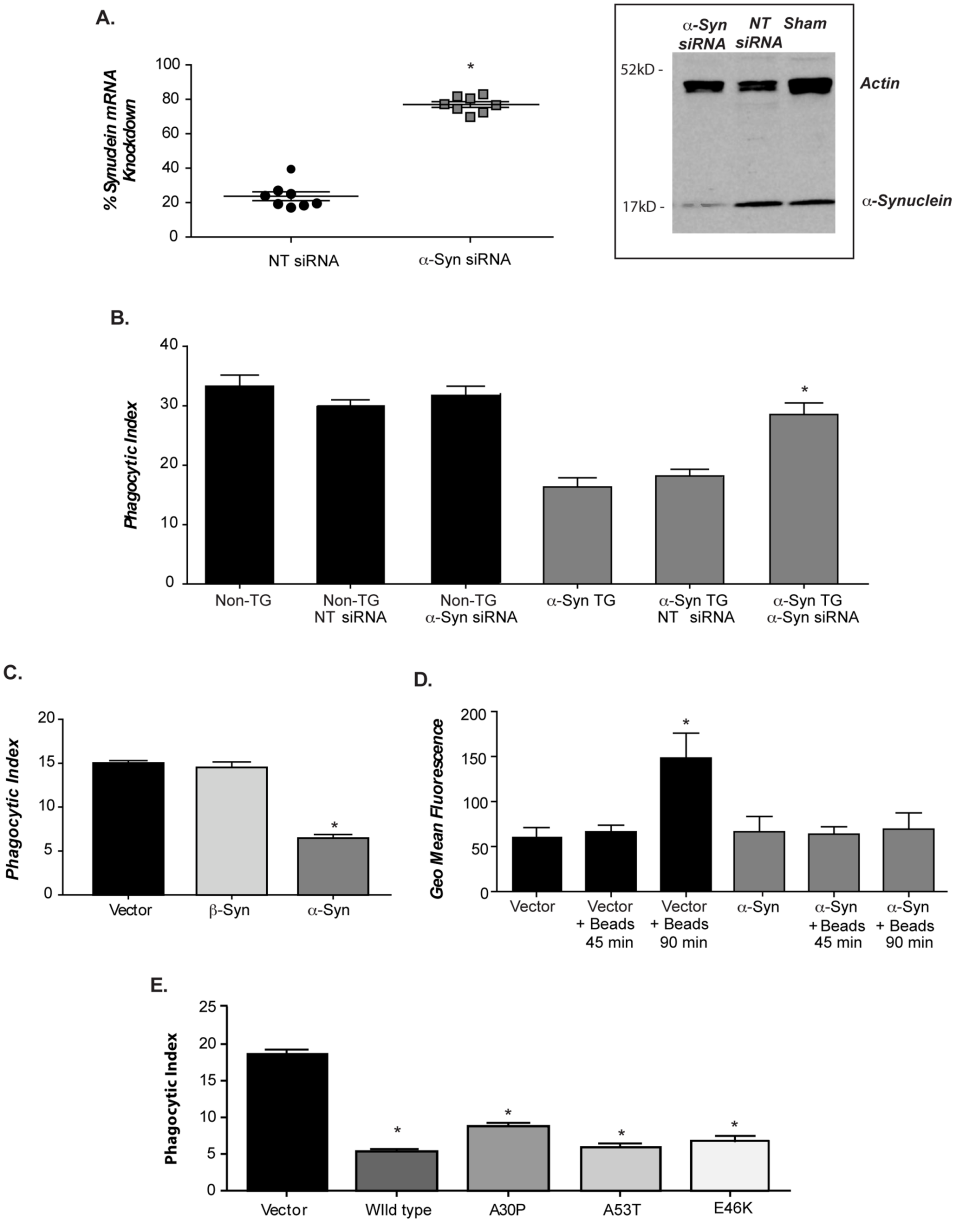
Alpha-synuclein drives decreased phagocytosis in α-syn BAC transgenic mice. (**A**) Peritoneal macrophages from line 26 mice were cultured in Accell media+/− human α-syn siRNA, or non- targeting siRNA. Human α-syn mRNA and protein levels were assessed by RT-PCR and immunoblot analysis (n = 2; 4 pups/GT/expt +/−s.e.m *p<0.01 when α-syn TG samples treated with α-syn siRNA were compared with α-syn TG or NT siRNA). (**B**) Following siRNA treatment macrophages were fed 10µ beads and a phagocytic index calculated (n = 2; 4 animals/GT/expt +/− s.e.m *p≤0.001 when the phagocytic index between α-syn TG microglia treated with α-syn siRNA and α-syn TG microglia alone or treated with NT siRNA were compared). (**C**) H4 cells were transfected with α- or β-syn expression vectors followed by addition of 4 µ beads and a phagocytic index calculated (n = 4 +/− s.e.m *p≤0.001 when the phagocytic index of α-syn transfected H4 cells was compared with vector of β-syn transfected cells). (**D**) α-syn or vector transfected H4 cells were fed beads followed by FM1-43 labeling and FACS analysis. Data is presented at geometric mean fluorescence (n = 4 +/− s.e.m *p≤0.001 when FM1-43 incorporation was compared between vector treated cells fed beads and cells transfected with α-syn fed beads). (**E**) H4 cells were transfected with wild type, A53T, A30P, or E46K α-syn expression vectors. After 2 days 4 µ beads were added for and the phagocytic index was measured (n = 4 +/− s.e.m *p≤0.05 when vector transfected cells were compared with cells transfected with wild type or the various familial mutations of α-syn).

To ensure that elevated levels of α-syn during development did not interfere with or alter expression of proteins necessary for phagocytosis, assays were performed in isolated peritoneal macrophages in which expression of the human transgene was knocked down with α-syn specific siRNA. Specific targeting of human α-syn with siRNA resulted in a 60–80% decrease in human α-syn mRNA (**Figure 5A** graph), which coincided with a concomitant decrease in α-syn protein levels at 72 hours (**Figure 5A** western). Targeted siRNA knockdown of human α-syn restored phagocytosis in α-syn TG macrophages while having minimal activity on non-TG cells (**Figure 5B**). These data reinforce the notion that elevated α-syn levels, rather than aberrant developmental changes are responsible for the observed phagocytic defects in the BAC transgenic animals.

Genetic manipulation of microglia is technically challenging since they do not transfect easily and are inclined to activation with transfection reagents. Therefore, we established a cultured cell model to assess the mechanism by which α-syn blocks vesicle dynamics. H4 cells are a human neuroglioma cell line [36] that are easy to transfect and can ingest latex beads (see below). Transient transfection of H4 cells with α- or β-syn resulted in elevated expression of these proteins, however only α-syn expression reduced phagocytosis (**Figure 5C**). Alpha-synuclein modulation of phagocytosis in H4 cells appeared to be due to alteration in vesicle trafficking, as vector transfected H4 cells displayed increased FM1-43 fluorescence following bead addition, while α-syn-transfected cells did not (**Figure 5D**). Importantly, the data generated in the transfected H4 system confirms the data from the α-syn genomic microglia and macrophages (compare Figure **3B** with Figure **5C**) since increased expression of α-syn in both systems prevented membrane expansion in response to bead stimulation and reduced phagocytosis by 50%.

Alpha-synuclein mutations found in familial PD have been associated with widespread α-syn pathology and cellular defects. To assess the effects of different PD-associated α-syn mutants on vesicle function, A53T, E46K and A30P α-syn constructs were transfected into H4 cells and all three mutations were found to significantly block phagocytosis as well as wild type α-syn when compared to vector transfected cells (**Figure 5E**) and this effect was dose-responsive to the level of α-syn expressed, with A53T and E46K having the greatest effect (data not shown).

### Alpha-synuclein is actively recruited to the phagocytic cup

**Figure 6:**
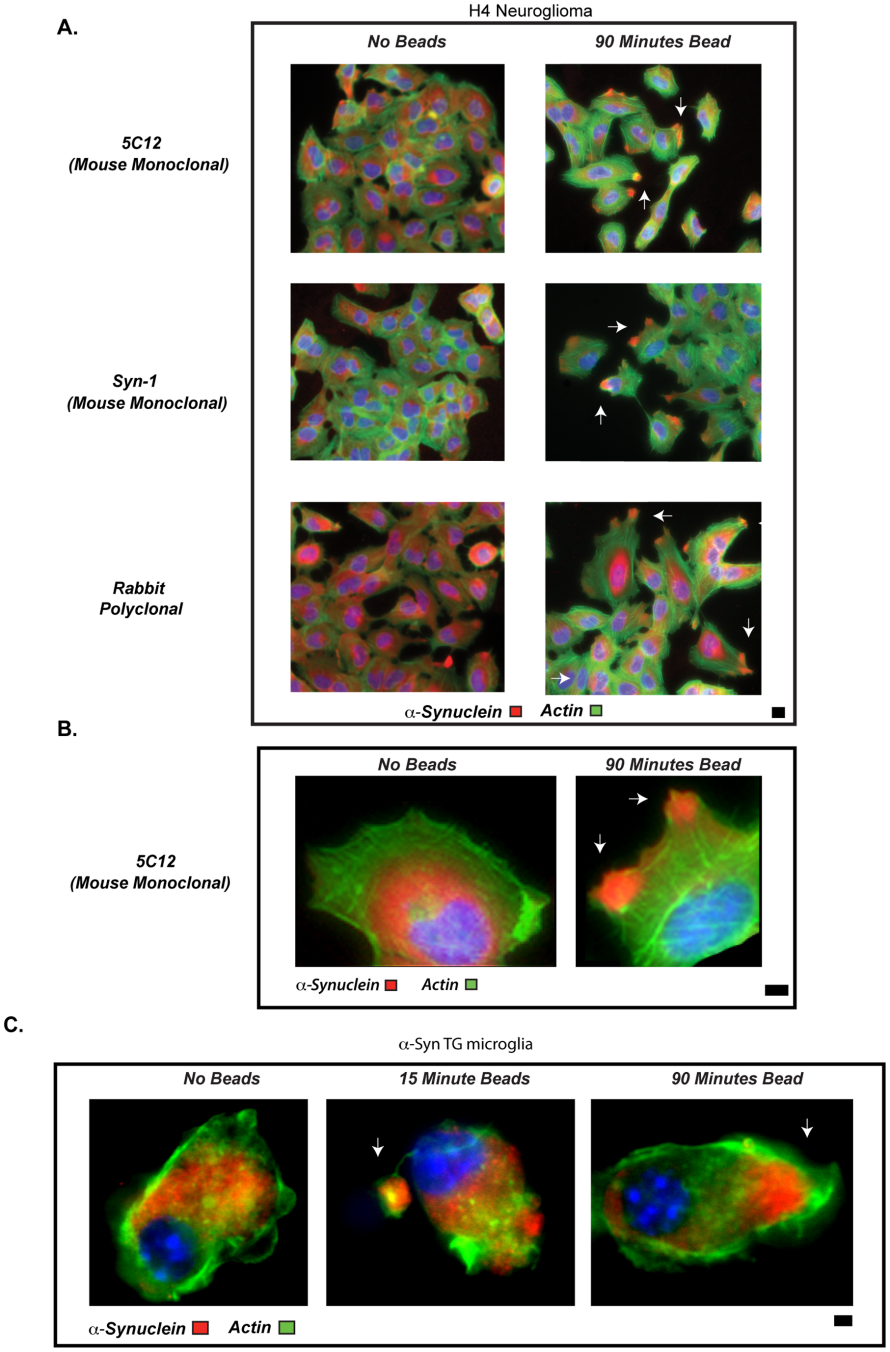
Alpha-synuclein translocates to the phagocytic cup. (**A**) H4 cells were stimulated with 4 µ beads for 0 or 90 minutes. Samples were stained with anti-α-syn antibodies: 5C12, Syn1 or a rabbit polyclonal (detected with secondary antibody, shown in red; see Methods for details of antibodies). Cells were counterstained with 488-phalloidin (green). Scale bars are equal to 1 micron. Arrows point to site of bead contact, and area of α-syn translocation, (beads are not visible by fluorescence; image is representative of n = 3). (**B**) Separate experiment from those in “A”, and shown at higher power. H4 cells were incubated +/− 4 µ beads for 90 minutes, and then stained for α-syn (red) with the antibody 5C12 (n = 5). Scale bars are equal to 1 micron. Arrows point to site of bead. (**C**) Line 3 α-syn TG microglia were incubated with 6µ beads for 0, 15, or 90 minutes. Samples were fixed, stained with an α-syn antibody (5C12; red) and counterstained with 488-phalloidin (green). Scale bars are equal to 1 micron. Arrows point towards site of bead contact (image is representative of n = 3).

Under resting conditions, with or without overexpression, α-syn is distributed throughout the cytoplasm of microglia and H4 cells (**Figure 6A**). To examine α-syn dynamics during the phagocytic process, H4 cells (expressing endogenous α-syn) were fixed at various times after bead addition, stained with anti-α-syn antibodies and localization of α-syn protein within the phagocytic cup assessed. Alpha-synuclein translocated to the point of bead contact in the phagocytic cup within 15macrophages (compare minutes, and by 90macrophages (compare minutes most of the α-syn had localized to this site (**Figure 6A** and **enlarged image 6B**). Visualization of translocation was independent of the antibody used since similar results were obtained with three unique α-syn antibodies (**Figure 6A**). Mobilization of α-syn to the phagocytic site was not restricted to the H4 system but also occurred in microglia after 15 and 90macrophages (compare minutes of bead addition (**Figure 6C**). This is the first example in which cellular distribution of α-syn can be altered by a specific stimulus, in a rapid timeframe. Furthermore, this distribution is reminiscent of the relatively consistent localization of α-syn in neuronal synapses.

### Inhibition of vesicle mobilization and fusion is associated with altered SNARE complex activity

**Figure 7:**
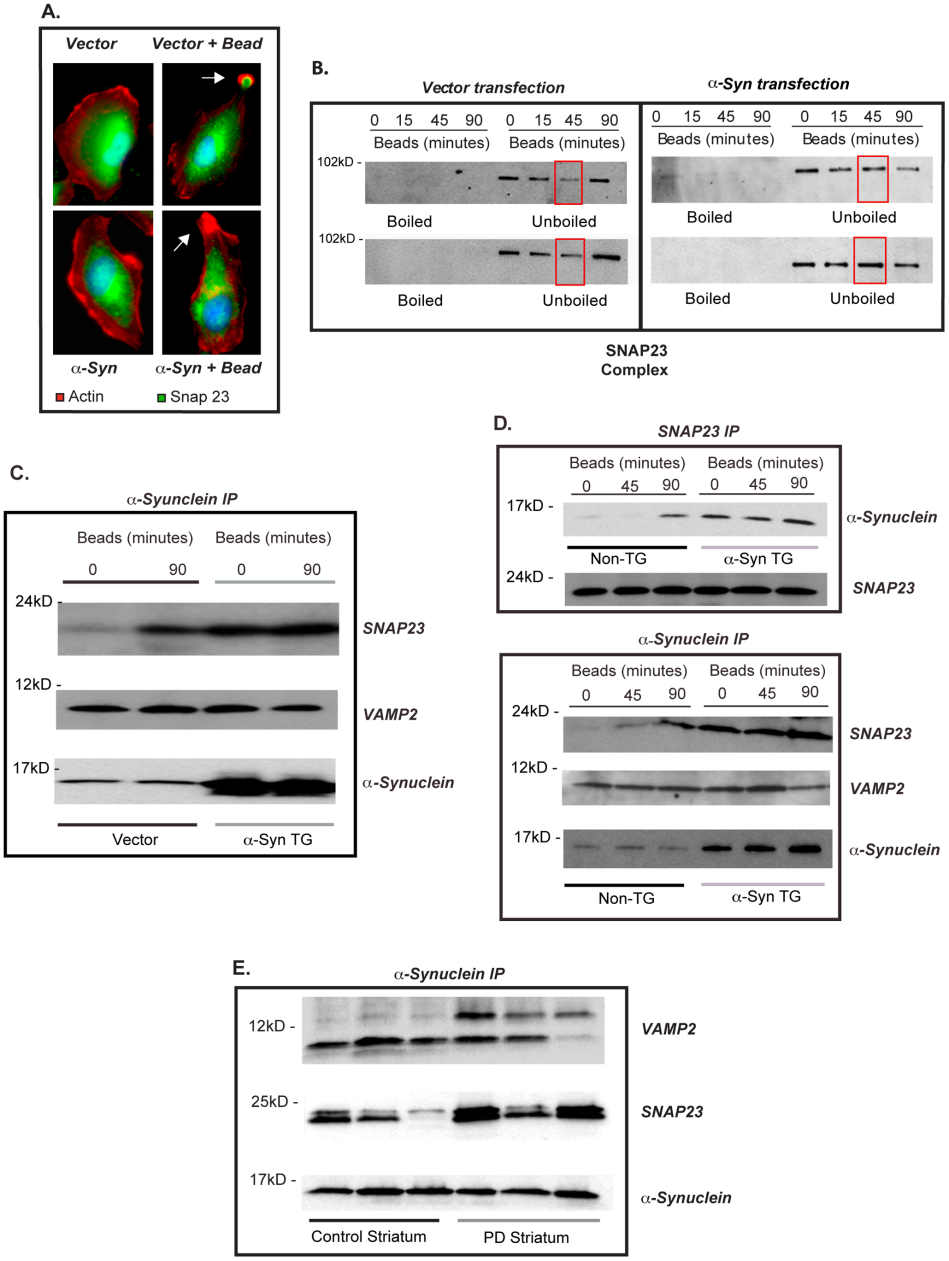
α-synuclein alters SNARE recycling. (**A**) H4 cells transfected with mock vector or α-syn and left unfed or fed 4 μ beads for 90 minutes, cells were processed for SNAP23 immunofluorescence (green), and polymerized actin (red.) Scale bars are equal to 1 micron image is representative of n = 3. Arrow points to area of bead contact and shows SNAP23 exclusion from the phagocytic cup with α-syn over expression. (**B**) Vector or α-syn transfected H4 cells were fed beads for various times. Cells were lysed and lysates were left unboiled or boiled at 100°C for 15 min. Samples were subjected to immunoblot analysis for SNAP23 containing SNARE complexes (two independent experiments are shown (top and bottom) (n = 3). (**C**) H4 cells transfected with α-syn or empty vector were fed beads and α-syn immunoprecipitated followed by immunoblotting for SNAP23 or VAMP2 (representative of n = 3). (**D**) Line 3 TG or non-TG microglia were fed and SNAP23 or α-syn immunoprecipitated followed by immunoblotting for α-syn, SNAP23, or VAMP2 respectively (n = 3) (E). Striatum from normal donors or PD patients were homogenized, α-syn immunoprecipitated followed by immunoblotting for SNAP23 or VAMP2. Three independent donors are shown here (representative of n = 2).

Published reports indicate that defective vesicle mobilization may be related to altered SNARE complex formation. SNARE components VAMP2 and SNAP23 are known to reside on vesicles. Immunofluorescence analysis of vector-transfected cells in our system, demonstrated that SNAP23 was recruited to the site of bead contact. Remarkably, with α-syn overexpression, SNAP23 remained in the cell body **(** **Figure 7A** **)**. Alpha-synuclein was recruited to the phagocytic cup in both endogenous and overexpressed conditions. These findings are consistent with the idea that α-syn is associated with SNARE function, and that over-expression of α-syn can interfere with normal SNARE activity. In this case, α-syn overexpression appeared to result in the exclusion of SNARE components from the site of bead contact and prevent membrane fusion.

To assess α-syn induced defects on SNARE complex assembly we employed the method described by Chen and Scheller [37], who demonstrated that SNARE proteins form energetically favorable, SDS stable complexes that can be identified by SDS-PAGE gel analysis. If samples are not boiled the SNARE complexes remain intact and run as a high molecular weight complex, however with sample boiling the complex is disrupted and the components run as individual proteins. H4 cells transfected with α-syn or control vector were stimulated with beads for 15, 45, or 90 minutes and SNAP23, as a component of SNARE complexes, was tracked by immunoblot analysis (**Figure 7B** **shows results from two independent experiments**). At time 0, SNAP23 could be visualized within a pre-existing high molecular weight SNARE complex (molecular weight of the complex in this figure is approximately 100 kDa). In control cells, addition of beads induced a decrease in SNAP23 containing SNARE complexes over a 45 minute time period, and by 90 minutes the high molecular weight complex containing SNAP23 was reformed. However, in α-syn overexpressing cells the pre-formed SNAP23 containing SNARE complex appeared to remain stable over the entire period of time (**Figure 7B**).

In order to test for association of α-syn with the SNARE complex in our system, we immunoprecipitated α-syn from H4 cells and looked for co-precipitation of SNAP23 and VAMP2. We found that endogenously expressed α-syn associated with SNAP23 during the progression of phagocytosis from 0 to 90 minutes (**Figure 7C**). In contrast, with α-syn overexpression, SNAP23 and α-syn were already associated at baseline (prior to bead addition) and the extent of this interaction did not change with phagocytic activity. Identical results were found in the microglia system with stimulation and α-syn overexpression (**Figure 7D**). SNAP23 association with α-syn was affected both by α-syn levels and cell stimulation, whereas α-syn binding to VAMP2 appeared unchanged over the course of the experiments (**Figure 7C and D**). Altered α-syn/SNARE interactions were also observed in the striatum of PD patients but not control subjects (**Figure 7E**).

### Cells from patients PD exhibit increased α-syn and defective phagocytosis

**Figure 8:**
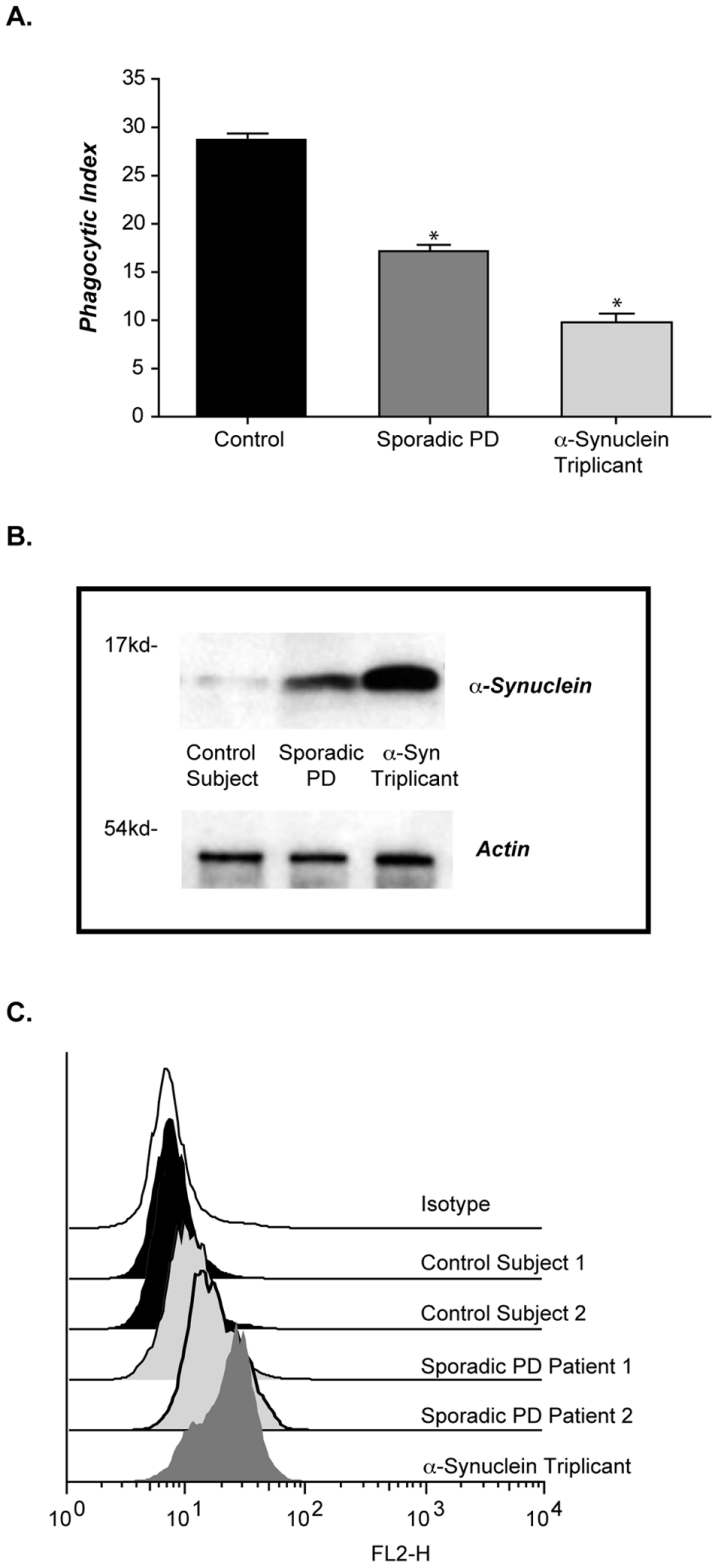
Fibroblasts isolated from PD patients display defective phagocytosis. (**A**) Fibroblast from 10 independent patients with sporadic PD, 10 age-matched normal donors, or an individual carrying the SNCA triplication were fed 1 μ beads and a phagocytosis index determined (n = 3+/− s.e.m. *p≤0.001 when the phagocytic index between the SNCA triplicant and sporadic PD patients fibroblasts were compared with the phagocytic index for control patient fibroblasts). (**B**) Fibroblasts from sporadic PD patient, age-matched control, and patient carrying the SNCA triplication were subject to immunoblot and flow cytometry analysis (image and histogram are representative of n = 3).

Parkinson's is a systemic disease, involving α-syn pathology and tissue dysfunction in multiple organs. Therefore, we asked if the phagocytic dysfunction characterized above could be used as a measure of systemic α-syn dysfunction in cells isolated from patients with PD. Although fibroblasts are not professional phagocytes, they are capable of engulfing small size particles. We compared fibroblasts isolated from a patient carrying the α-syn triplication (SNCA; [38], [39]) with fibroblasts from either 10 patients with sporadic PD or 10 age-matched control subjects. Fibroblasts isolated from the SNCA triplication patient had significantly reduced phagocytic activity (**Figure 8A**). Sporadic PD fibroblasts also demonstrated defective phagocytic activity, although the reduced activity was not as significant as the SNCA triplication case (**Figure 8A**). The defect in phagocytosis was associated with increased α-syn levels as assessed by immunoblot (**Figure 8B**) and intracellular staining (**Figure 8C**). Representative data from randomly selected donors are depicted here.

**Figure 9:**
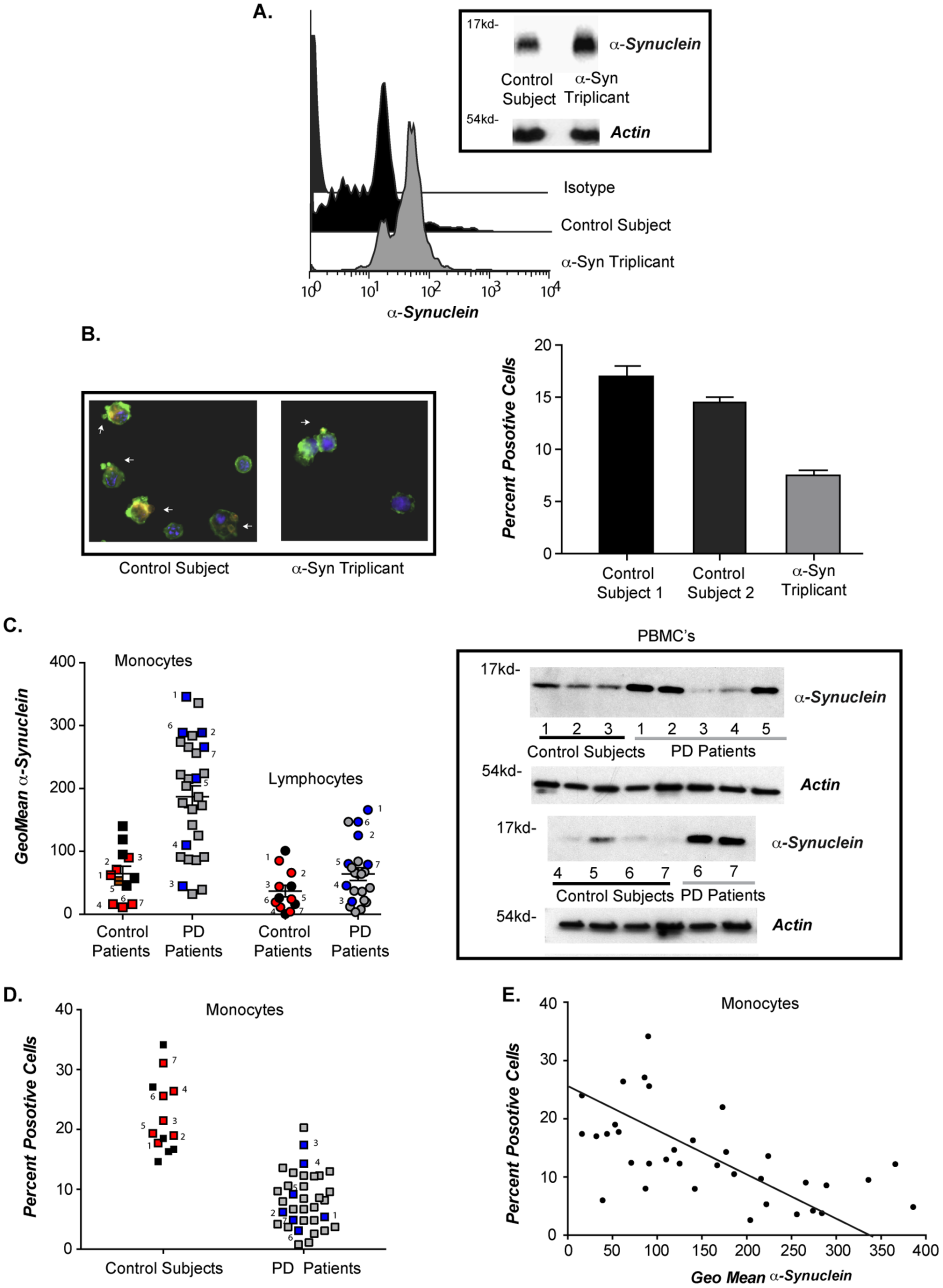
Monocytes isolated from PD patients display elevated α-syn levels and defective phagocytosis. (**A**) PBMCs isolated from a normal donor and an individual carrying the SNCA triplication were subject to immunoblot and flow cytometry analysis for α-syn levels (gel and histogram is representative of n = 1). (**B**) PBMCs from 2 normal donors and an individual carrying the SNCA triplication were fed fluorescently labeled RBCs. Percent monocytes positive for phagocytosis was determined by flow cytometry. Representative fluorescent images of 488 phalloidin stained PMBCs (green) with ingested RBCs (red) are shown. (**C**) PBMCs were stained for α-syn, and monocyte and lymphocyte populations were identified in the parent PBMC sample, using specific cell surface markers, we did not sort the cell populations. Intracellular α-syn levels are shown as the geo-mean of α-syn minus isotype control (n = 14 for normal donors and 34 sporadic PD +/− s.e.m *p≤001 when the α-syn geo mean for control donors was compared with the geo mean for sporadic PD PMBCs). PBMCs from 7 normal donors and 7 sporadic PD patients were subject to immunoblot analysis for α-syn. Specific PBMC samples 1–7 were selected for immunoblot analysis and marked in red for control donors and blue for sporadic PD donors. (**D**) Isolated PBMC's were fed fluorescently labeled RBCs. were subjected to flow cytometry analysis and percent monocytes positive for phagocytosis determine. Individual samples chosen for immunoblot analysis are marked out in red for control donors and blue for sporadic PD donors, (n = 14 for normal donors and 34 sporadic PD). The difference between the percent responses from control and PD was statistically significant with *p<0.001. (**E**). A correlation between α-syn levels, calculated as geo-mean and percent monocytes positive for phagocytosis was calculated.

While fibroblasts are a useful model system to assess phagocytic activity, monocytes isolated from patient blood could prove to be a more practical way to assess defective vesicle dynamics in large numbers of PD patients. Using immunoblotting and flow cytometry, we determined that monocytes isolated from a SNCA triplication patient expressed increased α-syn levels (**Figure 9A**). Phagocytic activity in monocytes isolated from a SNCA triplicate patient was compared to two normal control donors and the phagocytic activity was significantly reduced (**Figure 9B**).

To extend our findings to sporadic PD patients, peripheral blood mononuclear cells (PBMCs) isolated from thirty-four independent sporadic PD patients and fourteen age-matched controls, detailed patient data included in Table 1, were assessed for intracellular α-syn levels and phagocytic activity. Separate analysis of monocytic and lymphocytic populations within the PBMC samples was performed based on standard FACS forward and side-scatter parameters. Alpha-synuclein levels were elevated in monocytes and lymphocytes of sporadic PD patients when compared to controls (**Figure 9C**). Increased α-syn staining was confirmed by immunoblot analysis for a subset of the PBMC samples, as indicated by numbering (**Figure 9C**). No hemoglobin signal by immunoblot was detected, minimizing the possibility that RBC contamination influenced the data. These data indicate that α-syn is generally elevated in sporadic PD in peripheral white blood cells, and although the range of expression overlaps with control subjects, the high expressing patients are a clearly an identifiable population that may be of value for stratification in specific therapeutic intervention strategies. In addition to α-syn levels, we analyzed phagocytosis of fluorescently labeled CD47^−/−^ RBCs by sporadic PD patient monocytes, and a significant defect in phagocytosis was observed (**Figure 9D**). Although broad in range, defective phagocytosis significantly correlated with elevated intracellular α-syn levels (**Figure 9E**).

**Table 1:** Metadata of donors from control and PD blood used for phagocytosis and α-syn quantification.

PBMC Samples	*Sample Size*	*Age*	*Gender*	*Age of Onset*	*Age of Diagnosis*	*Duration*	*PBMC Count*
***Control***	14	66.62+/−7.14	M (5) F (9)	N/A	N/A	N/A	0.8 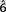 6+/−0.46
***PD***	34	67.06+/−9.08	M (18) F (16)	60.73+/−9.39	61.23+/−8.74	10+/−13.62	0.7 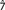 6+/−0.027

Demographic and disease progression information on control donor and PD patients who donated blood for our analysis of phagocytosis and intracellular α-syn levels.

## Discussion

Our data demonstrate that α-syn is expressed in innate immune cells and that the level of α-syn expression can regulate immune cell function. In a variety of experimental systems and human cells, we observe that increased α-syn dampens the ability of macrophages to clear cellular debris and release cytokines or chemokines. First, we demonstrate this effect in microglia and macrophages isolated from novel α-syn BAC transgenic mice that exhibit modest α-syn over expression via endogenous human α-syn gene promoter elements. The genomic mice results are corroborated by *in vitro* transfection experiments with H4 neuroglioma cells using stimulated phagocytic activity. We extended our findings to human cells through analysis of isolated monocytes and fibroblasts from a Parkinson's patient carrying a SNCA gene triplication, as well as from idiopathic PD patients. Similar to the SNCA triplication patients, idiopathic PD patients also exhibit elevated levels of α-syn, likely as part of the disease process. Finally, we confirmed our results with both macrophages and microglial cells in their respective *in vivo* settings, examining phagocytosis of beads, apoptotic cells, or CD47^−/−^ RBCs in living mice. We believe that all together, these consistent results have important implications for the role of α-syn in diseases such as PD, MSA, and Lewy body dementia, as well as in the synucleinopathies associated with peripheral organs such as the intestine and heart.

Our data suggest that increased levels of α-syn associated with aging and/or disease may lead to slower clearance of dead and dying cells, and may even impair clearance of α-syn itself, leading to a self-perpetuating cycle of increased α-syn that could accelerate disease progression. Furthermore, our findings may provide a new method for tracking the disease process in humans through the study of blood monocytes or skin fibroblast – cells that are easily accessible, and can be studied longitudinally from the same subjects. Finally, the phagocytic process provides a robust cell biological system for the study α-syn regulation of membrane vesicle events, and our results with SNARE complexes in this system are consistent with those of others studying neuronal systems.

Elevated α-syn is not limited to familial PD, but is also observed in sporadic PD [40]. However, publications reporting the expression of α-syn in microglial cells and macrophages are conflicting. Increased α-syn expression with age has been reported in macrophages; however, others have reported no expression at all [17], [41]. In the current report, we used culture systems that had minimal numbers of contaminating cells coupled with flow cytometry to specifically quantitate α-syn expression in cells isolated from mice as well as human subjects. Microglia isolated from α-syn TG mice expressed 2–3 fold higher levels of α-syn than those isolated from non-transgenic controls, as assessed by FACS analysis, and this result was confirmed by rt-PCR and immunoblot assessment. In human cells, we found increased levels of α-syn in triplication patients, suggesting our method of measurement was accurately quantitating α-syn levels in cells by flow cytometry, even when the differences in levels were not extraordinary.

Microglia are found throughout the brain with the highest density in the basal ganglia including the substantia nigra, olfactory telencephalon, and hippocampus, and all are areas that are affected in PD. This concentrated distribution may reflect a more direct role for microglia and inflammation in the development of PD than has been previously appreciated [42], and will likely be a fruitful area for future research. Innate immune cells are known to express familial PD genes including LRRK2, Parkin, DJ1, and GBA [43]–[45], and changes in expression of these proteins have been shown to have functional consequences in microglia. Loss of Parkin, for example, was shown to change inflammatory responses of microglia, both under basal and stimulated conditions, and was associated with dopamine neuron loss [43]. Additionally, inflammatory stimuli increase LRRK2 expression and induce LRRK2 phosphorylation in microglia. Interestingly, we have found that fibroblasts isolated from patients carrying mutations in proteins of familial PD genes other than α-syn also demonstrated altered phagocytosis (data not shown). It will be important to understand the functional consequence of familial PD-associated genes on microglia function and our data may offer an opportunity to identify common pathways for PD related to innate immune function, both in the CNS and the periphery.

Anomalies in phagocytosis and cytokine release have been documented with various autoimmune-related diseases (Crohn's Disease, COPD, Lupus, Sepsis), however the consequence of similar defects on neurodegenerative processes is yet not established. While inflammatory cytokines have been shown to be elevated in the brains of PD patients, cytokine levels are usually measured at the mRNA level or as total protein levels in brain extracts and therefore do not delineate between secreted and intracellular cytokines. Additionally, reports assessing peripheral inflammatory responses in PD patients have been varied, with reports showing increased cytokines in PD serum, but decreased release of cytokines from PBMCs isolated from PD patients [46]. In our *in vitro* system we demonstrate that elevated α-syn impairs the secretion of cytokines without altering inflammatory responses at the mRNA or total protein level, a defect that cannot be observed using pure analytical methodology since it cannot differentiate between intracellular and released cytokines. Misregulated cytokine release may cause chronically stressed and activated microglia, and contribute to perpetuating the inflammation and neurodegeneration seen in PD. Microglia release of critical neurotropic factors, such as NGF and BDNF [47], [48] and the general impact on vesicle secretion machinery by elevated levels of α-syn may be as important a contributor to disease as the alterations in inflammation.

Previous studies have identified defective mobilization of dopamine from the reserve vesicle pool in neurons of α-syn TG mice, and the culmination of numerous literature reports implicate α-syn as a negative regulator of vesicle trafficking/and or vesicle fusion [6]–[8], [25], [49], [50]. Although neuronal and inflammatory processes may appear distinct, the release of cytokine containing vesicles following an inflammatory insult is a process akin to the release of neurotransmitter containing synaptic vesicles following stimulation, utilizing similar SNARE machinery. Elevated α-syn levels have been shown to retard the release of neurotransmitters, an outcome that could lead to progressive neurodegeneration [50], and our observations for immunologic functions provide another avenue by which a common mechanism for decreased vesicular function may lead to neurodegeneration. We demonstrated that increased α-syn disrupts the release of inflammatory mediators from microglia and macrophages, as well as the recruitment of vesicular components necessary to support a phagocytic response. Normal phagocytic function was restored after α-syn siRNA treatment, demonstrating a direct role for α-syn in these processes. Furthermore, using cell culture systems we were able to induce a similar phagocytic deficit by increased α-syn expression in H4 cells.

Evoked cytokine release and phagocytosis are excellent model systems to study membrane and vesicle dynamics, as well as biological readouts of normal SNARE function. The reported relationship between α-syn and SNARE function has been mixed. Chandra et al. [51] demonstrated that α-syn overexpression rescued the lethality and neurodegeneration observed in CSP-1 knockout animals. The authors hypothesized that α-syn was protective because it could act as a SNARE chaperone in the absence of the normal chaperone, CSP-1. This hypothesis has recently been confirmed, and it was demonstrated that α-syn binds to members of the SNARE complex to facilitate SNARE assembly [25]. Alternatively, others have recently demonstrated thatα-syn binds to and sequesters arachadonic acid, the basis for the lipid platform that promotes SNARE complex formation and normal SNARE activity [26], [52]. In a different model system, wild type and A53T mutated α-syn were shown to block Golgi-ER trafficking, and the data suggested this was due to the interaction of α-syn with members of the ER/Golgi SNARE complex preventing the ability to form stable quaternary SNARE complexes [53]. The data in the work described here contains various aspects of these seemingly disparate observations.

In our experimental systems, we find that α-syn binds to members of the SNARE complex and specific SNARE interactions are dependent on cellular activity and α-syn levels. Increased interactions of α-syn and SNARE component translated to PD striatum, in addition, α-syn was found to interact with a unique higher molecular form of VAMP2 in PD tissue. This higher molecular weight VAMP2 could represent a novel post-translational modification and follow up experiments are required to identify the nature of this apparently modified VAMP2. Additionally, we observed alterations in preformed SNARE complexes, and recycling of SNARE complexes, in all systems with increased α-syn levels. Disassembly of these complexes is critical for continuous cycles of vesicle docking and fusion [54] and interference with proper disassembly may be a mechanism by which elevated levels of α-syn result in defective vesicle fusion. The SNARE interactions that we observed could be a means by which α-syn functions as a SNARE chaperone, alters SNARE complex formation, or sequesters SNARE complex components away from the site of activity. Importantly, our findings are consistent with those of others, indicating that α-syn expression is part of an intricate balance required for proper function of SNARE complexes and membrane vesicle activity. A key finding in our studies was the evoked recruitment of α-syn from the cytoplasm to the plasma membrane during a phagocytic stimulus. Increased recruitment of α-syn to the plasma membrane was correlated with decreased recruitment of SNARE complex components.

We observed elevated α-syn levels and defective phagocytosis in fibroblasts and monocytes isolated from SNCA triplication or sporadic PD patients, consistent with our cellular and *in vivo* mouse studies. Using intracellular flow cytometry to monitor α-syn levels, we determined that monocytes from PD patients have increased α-syn levels, which correlated with defective phagocytosis. Changes in phagocytosis did not correlate with patient age or duration of disease indicating that it is truly representative of intracellular α-syn levels. While the range in α-syn expression was broad, with overlap between PD patients and age-matched controls, these data indicate that a subpopulation of patients on the non-overlapping ends of the range can be identified for further study. Our observations were with a relatively small patient subset and will need to be extended and validated in a larger cohort as well as assessed in other synucleinopathies such as MSA and DLB. Such studies are important because CNS diseases are difficult to diagnose and usually neurodegeneration precedes observable symptomatic changes. Therefore, it is critical to identify peripheral markers that can be used as predictive biomarkers of CNS disease long before symptoms of degeneration arise. Overexpression of α-syn is a known risk factor for PD and understanding the development and timing of overexpression compared to onset of disease may provide insight into the disease process and provide ways to stratify patients for study of therapeutic intervention.

In summary, this paper provides 1) a new animal model to measure α-syn dysfunction both in the CNS and the PNS; 2) a cellular system to measure synchronized mobilization of α-syn and its functional interactions; 3) data regarding a potential role for innate immune cell function in the development and progression of PD; 4) an accessible peripheral biomarker to study and track these processes in human subjects.

## Methods

### Ethics Statement

All animal work was conducted according to relevant national and international guidelines. The human studies were performed following the study and protocol, which that The Parkinson's Institutional Review Board approved, and all subjects gave written informed consent for this study.

### Antibodies

Anti-alpha-synuclein antibodies used included Syn1 (Becton Dickinson Bioscience, Franklin Lakes, NJ), rabbit polyclonal from ThermoFisher Waltham MA (CA PA1-84490), and 5C12 and 9G5 (internally generated antibodies as described, [55]. Additional antibodies: SNAP23, VAMP2, (Synaptic Systems, Goettingen, Germany), Beta-synuclein (Epitomics, Burlingame, CA), FITC conjugated IBA, murine FITC IgG isotype control (BD Bioscience). 488-donkey anti-rabbit, 594-donkey anti-mouse and 594-donkey anti-rabbit, 488 and 594 phalloidin, DAPI, FM1-43FX, and fluorescent (excitation of 365 or 580) 4 or 10 μ beads and non-fluorescent 6 μ beads came from Life Science Technology (Carlsbad, CA). Rainbow™ Molecular Weight Markers and ECL plus came from GE Healthcare (Piscataway, NJ). LPS (LIST Biological Labs, Campbell, California). 4–20% gradient gels were purchased from Bio-Rad (Hercules, California). Saponin came from Sigma (St. Louis, MO).

### Animals

Bacterial Artificial Chromosome (BAC) clone RP11-458H10, containing the human SNCA gene sequence (Life Technologies, Carlsbad, CA) was modified to generate both the Rep1 mutation [56] and for lines 3 and 26 the E46K mutation [57] by BAC recombineering methods as described in Liu et al [58]. The mutations were confirmed by sequencing. Circular BAC constructs containing the hSNCA transgene (∼168 Kb) were used to perform pronuclear microinjection into B6SJL F2 mouse strains (The Jackson Laboratories, Bar Harbor, ME) in the concentration of 1–3 μg/μl followed by implantation into pseudo pregnant females (Xenogen Biosciences, Cranbury, NJ). Founders were identified by PCR genotyping using PCR primers on the 5′ end of the BAC: forward 5′-GATTTCCTTCTTTGTCTCCTATAGCACTGG-3′; reverse 5′-GAAGCAGGTATTACTGGCA GATGAGGC-3′; the middle of the BAC: forward 5′-GCCCTGTTTAGCAATCAACCTTCC-3′; reverse 5′-TACCTTGGAGTGAACCCTAAT CTGACTG-3′; the 3′ end of the BAC: forward 5′-GTGGTACACCGAGCAGTGGAAATGAG-3′; reverse 5′TGTCTGTTATCACCTTAAGTC TACTTTTGTCAGC-3′. Transgene copy number was determined by TaqMan qPCR method and determined as 4–5 copies. The TaqMan probe and primers for the hSNCA transgene for copy number determination were designed from 1.1 Kb exon 6 sequence (150–300 region). Probe (33 bp): FAM-CAC AAA GAC CCT GCT ACCATGTATTCACTTCAG-TAMARA. Primers: forward: 5′-AGTATCTGTACCTGCCCC CACTC-3′; reverse: 5′TGAAGCCACAAAATCCACAGC-3′. Founder animals were bred with B6D2F1 mice and maintained as heterozygotes on this background with non-TG littermates as controls. All mice were housed in a pathogen-free, climate controlled and given food and water ad libitum. All animal studies were reviewed and approved by the Institutional Animal Care and Use Committee at Élan pharmaceuticals in accordance with the National Institutes of Health Guide for the Care and Use of Laboratory Animals.

### Microglia cultures

Microglia were obtained from cerebral cortices of neonate mice (1–3 days old). Cortices were mechanically dissociated in Hanks' Balanced Salt Solution with 100 µg/ml DNAse I (Sigma). Dissociated cells were filtered through a 100-micron cell strainer, (Falcon, Heidelberg, Germany) and centrifugated at 200 g for 5 minutes. Pellets were resuspended in growth medium consisting of high glucose DMEM (Life Technologies), 10% FBS (Atlanta Biologicals, Lawrenceville, GA), and 25 ng/ml recombinant mouse granulocyte-monocyte colony stimulating factor, (rmGM-CSF, R&D Systems, Minneapolis, MN,) and each pup was cultured individually when performing heterozygote X heterozygote crosses which we followed by genotyping. Cells were plated at a density of two brains per T-75 plastic culture flask. After 7 days, the flasks were shaken at 200 rpm using a Lab-Line orbital shaker for 2 hr at 37°C. Cells suspensions were centrifuged at 200 g and re-suspended in growth medium.

### Immunoblotting

Briefly, microglia (5.0×10^5^ cells/well) were plated in a 24-well tissue culture plate and incubated overnight, cells were lysed in lysis buffer (CO-IP buffer and 1× Protease Inhibitor (Pierce, Rockford, IL)), resolved on 4–20% SDS-PAGE, and blotted to nitrocellulose membranes. For α-syn blots, the nitrocellulose membranes were boiled for 5 minutes in PBS. Membranes were probed with specific antibodies at 4°C overnight in 5% milk (Safeway Brand, Pleasanton, CA) and incubated with either horseradish peroxidase-conjugated anti-rabbit or anti-mouse secondary antibodies (Jackson ImmunoResearch, West Grove, PA) for 1 h at room temperature. The proteins were visualized by ECL plus according to the manufacturer's instructions. To confirm equal loading of proteins in each lane, the membranes were incubated in stripping buffer (62.5 mm Tris-HCl, pH 7.8, 100 mm β-mercaptoethanol, 2% SDS) for 30 min at 50°C and re-probed for actin (Sigma). To assess SNARE complexes samples were either boiled in Laemmle buffer for 15 minutes at 100°C or left un-boiled to assess SNARE complex formation.

### Intracellular α-syn staining

Microglia were fixed in 4% paraformaldehyde, then suspended in HBSS containing 2% FBS (Atlanta Biologicals,) at a concentration of 1×10^6^ cells per 100 μl. FcγRs were blocked using 100 ug/ml human IgG Fc-fragment (Calbiochem, Darmstadt, Germany). Cells were permeabilized with 0.5% Saponin (Sigma) for 30 minutes on ice and then incubated with 1 µg of primary antibody on ice for 60 minutes, cells were washed twice, and in the case of α-syn and the unlabeled murine isotype control, incubated with secondary antibody (1∶100 dilution) on ice for 30 minutes. Flow cytometry was performed using a FACScan cytometer (Becton Dickinson). Data were collected using Cellquest software (Becton Dickinson) and analyzed with FlowJo software (Tree Star, Ashland, OR).

### *In vitro* cytokine analysis

Microglia were cultured at 1×10^6^ cell/ml overnight followed by 18 hrs of LPS (100 ng/ml) stimulation. Supernatants were centrifuged to remove cellular debris and stored at −70°C until analyzed by ELISA. For intracellular cytokine analysis cells were treated with 1 μl Golgi-plug containing Brefeldin A (BD Biosciences) and LPS (100 ng/ml) for 6 hrs at which point supernatants were collected and the remaining cells lysed for intracellular cytokine analysis. Cytokines (TNF-α and IL6 were measured by ELISA (R&D Systems or Luminex multiplex analysis (Life Technologies)) as per the manufacture's protocol. For RNA level detection, microglia were stimulated for 6 hours with100 ng/ml LPS, cells were then snap frozen and mRNA levels assessed using QuantiGene 2.0 Plex Set Mouse, 12 plex (Panomics, Santa Clara, CA) as per the manufacturer's specification.

### *In vivo* cytokine secretion

The regimen of LPS injections for our studies was chosen based on published protocols which have been shown to trigger a chronic low level neuroinflammatory response [59]. Aged adult (6 month old) human α-syn overexpressing line 26 and wild-type littermate mice were given 7.5×1
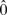
4 EU/kg from Escherichia coli O111: B4 (Sigma-Aldrich, Saint Louis, MO, USA) injections intraperitoneally (i.p.) twice a week for 6 months (n = 5 mice per sex, per genotype, per group), or remained un-dosed for 6 months (n = 5 mice per sex, per genotype, per group). After 6 months mice were sacrificed and serum assessed for cytokine levels by multiplex Elisa analysis as per the manufacture's instructions (Life sciences).

### Phagocytosis

Isolated line 26 non-TG and α-syn TG mouse microglia or macrophages from the mouse peritoneum (12 month old animals*)* were plated in 24-well plates at 1×10^5^ cells/well for 1 day. For phagocytosis assays using FluorSpheres particles, 5×1
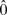
6 10 µ beads (Life Technologies) were washed with 1 ml of PBS 3 times to remove azide. Particles were then added (1∶10) to mouse microglia or macrophages for 90 minutes at 37°C. Unbound particles were washed away with PBS and cells were stained with Wright's Giemsa (Leukostat; Fisher) for 30 second for each stain and phagocytosis was visualized by light microscopy. Analysis was performed in a blinded investigator. For apoptotic cells, Jurkat T-cells were exposed to UV irradiation for 10∶minutes followed by overnight incubation. Induction of apoptosis was verified with annexin V/propidium iodide (Life Technologies) staining by flow cytometry. Apoptotic Jurkat T-cells cells were pelleted and resuspended at 1×1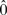/ml and added to microglia for 90∶minutes. Samples were processed as in the experiment with beads. The phagocytic index was calculated using the formula PI  =  (# particles ingested/#cells counted) ×100. 200 cells were visualized for each well and each condition was performed in duplicate and measured blindly.

### FM 1-43X labeling of membrane expansion

Experiments were carried out per Hackman et al. [22], with slight modifications. Microglia were plated on to a temperature sensitive UpCell plates (NUNC, ThermoFisher) overnight and followed by 10 µ fluorescent 365 bead addition the next day for 90 minutes. Cells were washed and treated with FM1-43× (1 mM) on ice for 1 minutes, cell were detached, washed 1× with cold PBS and bound FM1-43× fluorescence was quantified by flow cytometry using a FACScan cytometer (Becton Dickinson). Data were collected using Cellquest software (Becton Dickinson) and analyzed with FlowJo software (Tree Star). For H4 experiments, H4 cells obtained from ATCC (H4 (**ATCC® HTB-148™**) were plated onto 6 well UpCell plates (NUNC, Thermo Fisher), in 5 ml at 0.5 ml at 0.25×10^5^ cells. The next day cells were transfected with AMK vector or α-syn construct. 48 hrs later cells were fed 4 micron fluorescent 365 beads for 90 minutes and FM1-43× labeling performed as above.

### Isolation and labeling of CD47 knockout RBCs

Whole blood was collected from CD47 knockout mice via cardiac puncture. Red blood cells (RBCs) were isolated through a high-density solution (Lympholyte-M, Cedarlane) and labeled with green or red fluorescent lipophylic dye (PKH26GL, Sigma) that emits at a wavelength of 488 or 567 nm. The manufacture's protocol was followed.

### *In vivo* cell clearance

Experiment was performed as described [31]. Briefly the peritoneum's were lavaged 60 min after instillation of 10×1
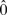
6 apoptotic Jurkat T-cells or PKH labeled CD47^−/−^ RBCs. Total cell counts were made for each mouse. For apoptotic Jurkat T-cell clearance a cytospin slide of lavaged cells was stained with modified Wright's Giemsa (Leukostat; Fisher) and phagocytosis was determined blinded by visual inspection. The phagocytic index was calculated using the formula PI  =  (# particles ingested/#cells counted) ×100 and at least 200 cells were counted/sample. For analysis of CD47^−/−^ RBC removal, lavages were spun at 1500 RPM for 5 minutes at 4°C and un-ingested RBCs lysed with 5 mls ACK lysis buffer (Life Technologies) for 5 minutes on ice. Cells were then cytospun and stained with DAPI (1∶10000) and phalloidin (1∶100) for 30 minutes, ingestion visualized by fluorescent microscopy and a phagocytic index calculated as above.

### Intracranial injections of CD47 KO RBCs labeled with PKH26

Experimental mice were anesthetized by intraperitoneal (IP) injection (SOP InVivo-012) of a Ketamine/Medotomidine cocktail (75 mg/kg +1 mg/kg respectively). During anesthesia, mice were placed within a Kopf stereotaxic frame equipped with a heating pad to avoid anesthesia-induced hypothermia. A 1-cm long mid-line scalp incision was made and 1 mm holes were drilled unilateral or bilateral into the skull using stereotaxic coordinates for the CA1 field of the hippocampus according to the Mouse Brain Atlas of Franklin and Paxinos, 1997. The animals in this study underwent one survival surgery comprising of a unilateral stereotaxic injection of 2 µl (∼5000 cells) of PKH labeled CD47-deficient RBC cells, diluted 1∶1000 in 1xPBS. Coordinates (x, y, z)  =  (−1.8, −2.3, −1.75) were relative to bregma. A depth of −1.85 was initially achieved to form a pocket for the injected red blood cells. Material was injected via a Hamilton syringe at a rate of 0.4 µl per min (2 µl total per site), with the needle untouched for 12 min prior to removal. Once the surgery was completed, the anesthetic effect of medetomidine was reversed by IP injection of atipamezole hydrochloride (1.0 mg/kg). Following surgery, the animals were returned to their home cage and observed until they recover from the anesthesia. The mice were checked at least once per day for local infection, bleeding, or open wounds, and for signs of distress or pain until euthanasia to perform tissue analysis. For tissue collection, mice were euthanized by C0_2_ narcosis, perfused intracardially with phosphate buffered saline, pH 7.4. The brain was subsequently extracted and post-fixed in 4% Paraformaldehyde in 0.1 M Phosphate Buffer, pH 7.4 for 48 hours. The tissue is transferred to PBS and stored in 4°C until sectioned.

#### Tissue Processing

Vibratome sections, 25 µm thick, from fixed brain samples were cut to reach injection site. Sections were counterstained with Hoechst for 5 minutes, rinsed three times with buffer, and cover-slipped.

#### Image Acquisition and Quantification

Digital images were captured using an Olympus BX61 microscope with Retiga EXi digital camera. Injection site (hippocampus) from tissue sections was imaged and visualized by combination of two channels (Nuclei/350 and RBCs/594). Integrated intensity (area x amount of fluorescence) signals were analyzed with MetaMorph imaging system (Molecular Devices).

### Assessment of kidney pathology

Kidney pairs were taken from 18 month old female line 26 non-TG littermate animals and 18 month old female heterozygote line 26 α-syn transgenic animals (n = 5 per genotype). Kidney pairs were immediately transferred to 50 ml of cold cryoprotectant (150 g sucrose, 200 ml 0.1 M PB, 150 ml Ethylene glycol, followed by 0.1 M PB to a final volume of 500 ml) and fresh tissue sat in above solution at 4°C until it sunk. Kidneys were then transferred to a mold containing OCT followed by freezing in liquid nitrogen (LN2). Frozen kidneys were sent to HistoTox Labs Inc (Boulder, CO) where staining for H&E, IgG, IgM, and complement C3 staining were performed as per the vendor specifications. Following antibody staining, extent and localization of staining for each kidney a certified pathologist scored pair blindly.

### siRNA knockdown experiments

Cultured peritoneal macrophages from 12 month old line 26 α-syn non-TG or TG mice were treated for 3 days with 1 µM pools of either human α-syn Accell siRNAs or non-target controls in Accell siRNA delivery media (Dharmacon Thermo Fisher). After 3 days, parallel cultures were used to measure human α-syn mRNA levels, protein levels, and phagocytic ability. Human α-syn mRNA levels were measured by TaqMan quantitative RT-PCR using the Applied Biosystems Gene Assay kit and the protocol provided by the supplier (Life Technologies). Human α-syn transcript levels were normalized relative to levels of the mouse housekeeping gene Beta-glucoronidase provide by the supplier. Human α-syn transcript levels were normalized relative to transcript levels of the mouse housekeeping gene Beta-glucoronidase.

### H4 transfection experiments

H4 neuroglioma cells obtained from ATCC, (**H4 (ATCC® HTB-148™**), were plated in 0.5 ml at 0.25×10^5^ cells per ml overnight followed by transfection with 100 ng DNA using 1 μl Lipofectamine 2000 (Life Science) per well and transfection was performed according to manufacturer's protocol. Plasmids used included AGMK vector control, AMK vector control for fluorescent studies, AGMKα-syn wildtype, AGMKα-syn E46K mutation, AGMKα-syn A30P mutation, or AGMK α-syn A53T mutation. The next day medium was aspirated and replaced with fresh DMEM/10% FBS. Experiments were performed 48 hours after transfection and transfection efficiency was found to be 80% as determined by GFP positive cells, increase in 5C12 staining, and protein expression by immunoblot.

### Microscopy analysis

For α-syn translocation analysis H4 cells or line 3 α-syn TG microglia were plated at 25,000 cells in a 24 well plate overnight at which point cells were fed 4μ or 6 micron non-fluorescent beads for 0, 45, or 90 minutes. Cells were fixed with 4% PFA for 20 minutes, and then permeabilized with 0.5% saponin (Sigma) and blocked with 1% BSA for 30 minutes on ice. For microglia experiments, Fc-receptors were blocked with 100 ug/ml human Fc-fragment for 30 minutes prior to addition of 1 µg of primary antibody 5C12 which was added overnight at 4°C, cells were then washed twice and incubated with 488 conjugated secondary antibody (1∶100 dilution) for 30 minutes. Cell were washed twice in PBS and then stained for actin with 1∶100 of 488 phalloidin and the nuclear stain DAPI at 1∶1000 for 30 minutes. Cells were visualized on an Olympus1×81 scope with a 20×/0.45 Ph1 objective or a 63×/0.70 ph2 objective. Images were taken using MetaMorph software. SNAP23 visualization was performed similarly except H4 cells were transiently transfected with 100 ng of vector, α-syn, or the delta119 form of α-syn for 24 hours at which point cells were unchallenged or fed beads for 90 beads. Cells were washed and fixed in 4% PFA for 20 minutes followed by permeabilization and blocking as above. Anti-SNAP23 was added at 1∶100 overnight at 4°C. Cells were washed and stained with anti-rabbit 488 at 1∶100 for an hour followed by 2 washes in PBS and then cells were incubated with 594 phalloidin at 1∶100 and DAPI at 1∶1000. Images were collected as above. Scale bars are equal to 1 micron for all images.

### Immunoprecipitations

Line 26 α-syn non-TG or TG microglia were plated overnight at 1×1
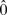
6 cell/ml. Cells were then fed 10 µ beads for 45 or 90 minutes. Cells were washed 3X in PBS and then lysed on ice for 10 minutes with CO-IP buffer (Pierce) containing Halt protease/phosphatase inhibitor (Pierce). Lysates were transferred to tubes containing Protein G agarose beads and lysates were pre-cleared for 30 minutes at 4°C with rotation. Lysates were spun at 14,000 rpm for 5 minutes and were transferred to new tubes containing Protein G agarose and 1ug 9G5 or 1ug anti-SNAP23. Lysates were incubated with antibody at 4°C overnight with rotation followed by washing the next day with 2X Sten buffer followed by 2 subsequent washes with 1X Sten buffer. Agarose beads were taken up in 1X Laemmli buffer, boiled and immunoblot performed as above.

Co-IP experiment in H4 cells were performed essentially identically except H4 cells were plated at 0.5×1
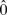
6 followed by vector or α-syn transfection for 48 hrs at which point 4 micron beads were added and CO-IP analysis performed.

### Skin biopsies

One four mm skin punch biopsy was taken per patient using a standard punch biopsy technique. All biopsies were taken from the upper inner arm, an area that is mostly unexposed to direct sunlight. The study and protocol had Institutional Review Board approval from The Parkinson's Institute and all subjects gave written informed consent for this study.

### Fibroblast cell culture

Primary fibroblasts from biopsies were derived from standard skin explant cultures and were banked at low passage numbers. Fibroblasts were cultured in Dulbecco's minimum essential medium, high-glucose (DMEM) with 10% fetal bovine serum, 100 IU/ml penicillin, 100 μg/ml streptomycin, 200 mM glutamine, and 10 mM non-essential amino acids (all purchased from Invitrogen, Carlsbad, CA). Experiments were performed with 1υ 488 fluorescent beads fed for 2 hrs when the cells were at passages 6–12.

### PBMC Isolation, phagocytosis and intracellular α-syn assessment

Inclusion criteria for this study were as follows; diagnosis of idiopathic PD for at least 2 years by movement disorder specialist at TPI, age at recruitment, 50–80 yrs, inclusion of both males and females. Biomarker consent form ECH-10–17 was signed and **c**linical/neurological assessment (including UPDRS and/or Hoehn and Yahr), MoCA (from chart or perform during visit), B-SIT, and smoking history/questionnaire obtained. Blood samples from PD patients and control individuals were collected by the Parkinson Institute in 2× citrate tubes, 4.5 ml (Cat. No. 369714) in the morning of and delivered to Élan in a blinded fashion each afternoon. Upon arrival, samples were spun at 3000 rpm for 30 min without break to remove the plasma and eliminate platelets. PBMCs were then isolated from donor using BD Vacutainer tubes. Isolated PBMCs were washed with PBS/Citrate buffer (1.49 mM Na2H2PO4, 9.15 mM Na2HPO4, 139.97 mM NaCl, 13 mM C6H5Na3O7, pH 7.2) to diminish platelet contamination. PBMCs were counted using a hemocytometer, and resuspended at 1×10^6^/ml in culture media (DMEM/10% FBS (Altanta Biologics)/Pen-strep/L-Glutamine). 1×1
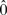
6 cells were plated in duplicate in a 6-well Upcel plate (NUNC) and cultured at 37°C, 10%CO2 for 1 hr at which point PKH red labeled RBCs at were added at a RBC to PBMCs ratio of 5:1 for 1.5 hrs. Plates were placed on ice for 10 minutes to stop the phagocytic activity and detach the PBMCs, cells were then collected and spun down the cells at 800 g for 10 min at 4°C. 1 ml of ACK RBC lysing buffer was added followed by gentle vortexing to break down the RBCs outside the cells. After 1 minute of incubation, cells were spun at 800 g, 4°C for 10 minutes, buffer aspirated, and cells fixed with 2% PFA at 4°C for at least 60 minutes. Cells were then spun down and resuspended in 300 µL cold PBS and percent monocytes ingesting PKH labeled RBCs assessed by flow cytometry using a FACScan cytometer (Becton Dickinson). Data were collected using Cellquest software (Becton Dickinson) and analyzed with FlowJo software (Tree Star). Mouse microglia cells were included in each assay as an intra-assay control.

Intracellular α-syn staining was performed on 1×1
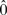
6 PBMCs. Cells were spun down and fixed using PBS/2% PFA at 4°C for 1 hr, then washed with PBS and resuspended in 0.5 ml PBS. Cells were permeablized and Fc receptors blocked using Cell Permeable Buffer (Thermo Fisher) and human fc blocking reagent at 4°C for 1 hr. Cells were then split into a 96-well V-bottom plate. msIgG2b or 5C12, was added at 10 µg/ml final concentration, and samples incubated at RT for 1 hr in the presence of the Cell Permeable buffer (Thermo Fisher) with human Fc blocking reagent. Cells were spun down washed with PBS/1% FBS once, and incubated with anti-msIgG (Fc)-PE at 1∶100 at 4°C for 30 minutes. Cells were then spun down, washed with PBS/1% FBS 2X, and mean fluorescence determined by flow cytometry using a LSRII using Facs Diva software (BD Biosystems) and analysis performed using FlowJo (Tree Star).

### Statistics

Data are presented as mean ± SEM. Statistical analysis was performed using two-tailed Student's *t*-test of unpaired samples. For multiple comparisons data were evaluated by ANOVA with *post hoc* analysis by the two-tailed Dunnetts test. Statistical analyses for human PBMC samples were performed using a two-factor nested design model (Neter, Wasserman and Kutner 1985). The two factors were a fixed effect of Group to compare control and PD, and a random effect of patient id. The results of 2 runs were replicates and were averaged. Normality and homogeneity of variance were examined by the Shapiro-Wilk test [60] and the Levene test [61] at the 0.01significance level, respectively. When either assumption was significant, the statistical analysis was performed on suitably transformed data. All statistical analyses were conducted using JMP 8.0 (SAS Institute Inc. 2008). Significant differences were defined as p<0.05.

## Supporting Information

Figure S1 Microglia isolated from line 26/syn ^null^ or α-syn ^null^ littermates were stimulated with LPS in the presence or absence of Brefeldin A. Tissue culture supernatant was assessed for TNF-α production by ELISA (n = 2; 5 pups/GT/expt +/− s.e.m *p≤0.001 when α-syn TG samples were compared with non-TG).
(EPS)

## References

[1] Fink AL (2006) The aggregation and fibrillation of alpha-synuclein. Accounts of Chemical Research 39: 628–634. 16981679 10.1021/ar050073t

[2] Uversky VN (2008) Alpha-synuclein misfolding and neurodegenerative diseases. Current Protein & Peptide Science 9: 507–540. 18855701 10.2174/138920308785915218

[3] Paleologou KE, Irvine GB, El-Agnaf OM (2005) Alpha-synuclein aggregation in neurodegenerative diseases and its inhibition as a potential therapeutic strategy. Biochemical Society Transactions 33: 1106–1110. 16246056 10.1042/BST20051106

[4] Schiesling C, Kieper N, Seidel K, Kruger R (2008) Review: Familial Parkinson's disease–genetics, clinical phenotype and neuropathology in relation to the common sporadic form of the disease. Neuropathology & Applied Neurobiology 34: 255–271. 18447897 10.1111/j.1365-2990.2008.00952.x

[5] Lucking CB, Brice A (2000) Alpha-synuclein and Parkinson's disease. Cellular & Molecular Life Sciences 57: 1894–1908. 11215516 10.1007/PL00000671 PMC11146993

[6] Nemani VM, Lu W, Berge V, Nakamura K, Onoa B, et al (2010) Increased expression of alpha-synuclein reduces neurotransmitter release by inhibiting synaptic vesicle reclustering after endocytosis. Neuron 65: 66–79. 20152114 10.1016/j.neuron.2009.12.023 PMC3119527

[7] Kim KS, Park J-Y, Jou I, Park SM (2010) Regulation of Weibel-Palade Body Exocytosis by Î±-Synuclein in Endothelial Cells. Journal of Biological Chemistry 285: 21416–21425. 20448034 10.1074/jbc.M110.103499 PMC2898423

[8] Auluck PK, Caraveo G, Lindquist S (2010) -Synuclein: membrane interactions and toxicity in Parkinson's disease. Annual Review of Cell & Developmental Biology 26: 211–233. 10.1146/annurev.cellbio.042308.113313 20500090

[9] Thayanidhi N, Helm JR, Nycz DC, Bentley M, Liang Y, et al (2010) {alpha}-Synuclein Delays Endoplasmic Reticulum (ER)-to-Golgi Transport in Mammalian Cells by Antagonizing ER/Golgi SNAREs. Mol Biol Cell 21: 1850–1863. 20392839 10.1091/mbc.E09-09-0801 PMC2877643

[10] Ikemura M, Saito Y, Sengoku R, Sakiyama Y, Hatsuta H, et al (2008) Lewy body pathology involves cutaneous nerves. J Neuropathol Exp Neurol 67: 945–953. 18800013 10.1097/NEN.0b013e318186de48

[11] Reichmann H (2011) View point: etiology in Parkinson's disease. Dual hit or spreading intoxication. J Neurol Sci 310: 9–11. 21600591 10.1016/j.jns.2011.04.016

[12] Jellinger KA (2011) Synuclein deposition and non-motor symptoms in Parkinson disease. J Neurol Sci 310: 107–111. 21570091 10.1016/j.jns.2011.04.012

[13] Wakabayashi K, Mori F, Tanji K, Orimo S, Takahashi H (2010) Involvement of the peripheral nervous system in synucleinopathies, tauopathies and other neurodegenerative proteinopathies of the brain. Acta Neuropathol 120: 1–12. 20532896 10.1007/s00401-010-0706-x

[14] Halliday GM, Stevens CH (2011) Glia: initiators and progressors of pathology in Parkinson's disease. Mov Disord 26: 6–17. 21322014 10.1002/mds.23455

[15] Lee SJ (2008) Origins and effects of extracellular alpha-synuclein: implications in Parkinson's disease. J Mol Neurosci 34: 17–22. 18157654 10.1007/s12031-007-0012-9

[16] Roodveldt C, Labrador-Garrido A, Gonzalez-Rey E, Fernandez-Montesinos R, Caro M, et al (2010) Glial innate immunity generated by non-aggregated alpha-synuclein in mouse: differences between wild-type and Parkinson's disease-linked mutants. PLoS ONE 5: e13481. 21048992 10.1371/journal.pone.0013481 PMC2964342

[17] Austin SA, Floden AM, Murphy EJ, Combs CK (2006) Alpha-synuclein expression modulates microglial activation phenotype. J Neurosci 26: 10558–10563. 17035541 10.1523/JNEUROSCI.1799-06.2006 PMC6674709

[18] Erwig LP, Henson PM (2007) Immunological consequences of apoptotic cell phagocytosis. Am J Pathol 171: 2–8. 17591947 10.2353/ajpath.2007.070135 PMC1941587

[19] Elliott MR, Ravichandran KS (2010) Clearance of apoptotic cells: implications in health and disease. J Cell Biol 189: 1059–1070. 20584912 10.1083/jcb.201004096 PMC2894449

[20] Braun V, Niedergang F (2006) Linking exocytosis and endocytosis during phagocytosis. Biol Cell 98: 195–201. 16480341 10.1042/BC20050021

[21] Palokangas H, Mulari M, Vaananen HK (1997) Endocytic pathway from the basal plasma membrane to the ruffled border membrane in bone-resorbing osteoclasts. Journal of Cell Science 110: 1767–1780. 9264464 10.1242/jcs.110.15.1767

[22] Hackam DJ, Rotstein OD, Sjolin C, Schreiber AD, Trimble WS, et al (1998) v-SNARE-dependent secretion is required for phagocytosis. Proceedings of the National Academy of Sciences 95: 11691–11696. 10.1073/pnas.95.20.11691 PMC21702 9751727

[23] Huynh KK, Kay JG, Stow JL, Grinstein S (2007) Fusion, Fission, and Secretion During Phagocytosis. Physiology 22: 366–372. 18073409 10.1152/physiol.00028.2007

[24] Murray RZ, Kay JG, Sangermani DG, Stow JL (2005) A Role for the Phagosome in Cytokine Secretion. Science 310: 1492–1495. 16282525 10.1126/science.1120225

[25] Burré J, Sharma M, Tsetsenis T, Buchman V, Etherton MR, et al (2010) Alpha-Synuclein Promotes SNARE-Complex Assembly in Vivo and in Vitro. Science 329: 1663–1667. 20798282 10.1126/science.1195227 PMC3235365

[26] Darios F, Ruiperez V, Lopez I, Villanueva J, Gutierrez LM, et al (2010) [alpha]-Synuclein sequesters arachidonic acid to modulate SNARE-mediated exocytosis. EMBO Rep 11: 528. 20489724 10.1038/embor.2010.66 PMC2897113

[27] Kuo YM, Li Z, Jiao Y, Gaborit N, Pani AK, et al (2010) Extensive enteric nervous system abnormalities in mice transgenic for artificial chromosomes containing Parkinson disease-associated alpha-synuclein gene mutations precede central nervous system changes. Hum Mol Genet 19: 1633–1650. 20106867 10.1093/hmg/ddq038 PMC2850613

[28] Burré J, Sharma M, Tsetsenis T, Buchman V, Etherton MR, et al (2010) Alpha-Synuclein Promotes SNARE-Complex Assembly in Vivo and in Vitro. Science 329: 1663–1667. 20798282 10.1126/science.1195227 PMC3235365

[29] Gu XL, Long CX, Sun L, Xie C, Lin X, et al (2010) Astrocytic expression of Parkinson's disease-related A53T alpha-synuclein causes neurodegeneration in mice. Mol Brain 3: 12. 20409326 10.1186/1756-6606-3-12 PMC2873589

[30] Norsworthy PJ, Fossati-Jimack L, Cortes-Hernandez J, Taylor PR, Bygrave AE, et al (2004) Murine CD93 (C1qRp) Contributes to the Removal of Apoptotic Cells In Vivo but Is Not Required for C1q-Mediated Enhancement of Phagocytosis. The Journal of Immunology 172: 3406–3414. 15004139 10.4049/jimmunol.172.6.3406

[31] Stuart LM, Takahashi K, Shi L, Savill J, Ezekowitz RAB (2005) Mannose-Binding Lectin-Deficient Mice Display Defective Apoptotic Cell Clearance but No Autoimmune Phenotype. The Journal of Immunology 174: 3220–3226. 15749852 10.4049/jimmunol.174.6.3220

[32] Mukundan L, Odegaard JI, Morel CR, Heredia JE, Mwangi JW, et al (2009) PPAR-[delta] senses and orchestrates clearance of apoptotic cells to promote tolerance. Nat Med 15: 1266–1272. 19838202 10.1038/nm.2048 PMC2783696

[33] Gardai SJ, McPhillips KA, Frasch SC, Janssen WJ, Starefeldt A, et al (2005) Cell-surface calreticulin initiates clearance of viable or apoptotic cells through trans-activation of LRP on the phagocyte. Cell 123: 321–334. 16239148 10.1016/j.cell.2005.08.032

[34] Rőszer T, Menéndez-Gutiérrez MP, Lefterova MI, Alameda D, Núñez V, et al (2011) Autoimmune Kidney Disease and Impaired Engulfment of Apoptotic Cells in Mice with Macrophage Peroxisome Proliferator-Activated Receptor γ or Retinoid X Receptor α Deficiency. The Journal of Immunology 186: 621–631. 21135166 10.4049/jimmunol.1002230 PMC4038038

[35] Munoz LE, Lauber K, Schiller M, Manfredi AA, Herrmann M (2010) The role of defective clearance of apoptotic cells in systemic autoimmunity. Nat Rev Rheumatol 6: 280–289. 20431553 10.1038/nrrheum.2010.46

[36] Outeiro TF, Putcha P, Tetzlaff JE, Spoelgen R, Koker M, et al (2008) Formation of Toxic Oligomeric α-Synuclein Species in Living Cells. PLoS ONE 3: e1867. 18382657 10.1371/journal.pone.0001867 PMC2270899

[37] Chen YA, Scheller RH (2001) SNARE-mediated membrane fusion. Nat Rev Mol Cell Biol 2: 98–106. 11252968 10.1038/35052017

[38] Singleton AB, Farrer M, Johnson J, Singleton A, Hague S, et al (2003) alpha-Synuclein locus triplication causes Parkinson's disease. Science 302: 841. 14593171 10.1126/science.1090278

[39] Carr J, de la Fuente-Fernandez R, Schulzer M, Mak E, Calne SM, et al (2003) Familial and sporadic Parkinson's disease usually display the same clinical features. Parkinsonism Relat Disord 9: 201–204. 12618054 10.1016/s1353-8020(02)00048-2

[40] Tan EK, Kwok HH, Tan LC, Zhao WT, Prakash KM, et al (2010) Analysis of GWAS-linked loci in Parkinson disease reaffirms PARK16 as a susceptibility locus. Neurology 75: 508–512. 20697102 10.1212/WNL.0b013e3181eccfcd PMC2918477

[41] Brighina L, Prigione A, Begni B, Galbussera A, Andreoni S, et al (2010) Lymphomonocyte alpha-synuclein levels in aging and in Parkinson disease. Neurobiology of Aging 31: 884–885. 18676060 10.1016/j.neurobiolaging.2008.06.010

[42] Tansey MG, Goldberg MS (2010) Neuroinflammation in Parkinson's disease: its role in neuronal death and implications for therapeutic intervention. Neurobiol Dis 37: 510–518. 19913097 10.1016/j.nbd.2009.11.004 PMC2823829

[43] Solano RM, Casarejos MJ, Menendez-Cuervo J, Rodriguez-Navarro JA, Garcia de Yebenes J, et al (2008) Glial dysfunction in parkin null mice: effects of aging. J Neurosci 28: 598–611. 18199761 10.1523/JNEUROSCI.4609-07.2008 PMC6670347

[44] van Horssen J, Drexhage JA, Flor T, Gerritsen W, van der Valk P, et al (2010) Nrf2 and DJ1 are consistently upregulated in inflammatory multiple sclerosis lesions. Free Radic Biol Med 49: 1283–1289. 20673799 10.1016/j.freeradbiomed.2010.07.013

[45] Miklossy J, Arai T, Guo JP, Klegeris A, Yu S, et al (2006) LRRK2 expression in normal and pathologic human brain and in human cell lines. J Neuropathol Exp Neurol 65: 953–963. 17021400 10.1097/01.jnen.0000235121.98052.54 PMC7185781

[46] Hasegawa Y, Inagaki T, Sawada M, Suzumura A (2000) Impaired cytokine production by peripheral blood mononuclear cells and monocytes/macrophages in Parkinson's disease. Acta Neurol Scand 101: 159–164. 10705937 10.1034/j.1600-0404.2000.101003159.x

[47] Scalzo P, Kummer A, Bretas TL, Cardoso F, Teixeira AL (2010) Serum levels of brain-derived neurotrophic factor correlate with motor impairment in Parkinson's disease. J Neurol 257: 540–545. 19847468 10.1007/s00415-009-5357-2

[48] Nagatsu T, Mogi M, Ichinose H, Togari A (2000) Changes in cytokines and neurotrophins in Parkinson's disease. J Neural Transm Suppl: 277–290. 10.1007/978-3-7091-6301-6_19 11205147

[49] Lotharius J, Brundin P (2002) Impaired dopamine storage resulting from alpha-synuclein mutations may contribute to the pathogenesis of Parkinson's disease. Hum Mol Genet 11: 2395–2407. 12351575 10.1093/hmg/11.20.2395

[50] Lotharius J, Brundin P (2002) Pathogenesis of Parkinson's disease: dopamine, vesicles and alpha-synuclein. Nat Rev Neurosci 3: 932–942. 12461550 10.1038/nrn983

[51] Chandra S, Gallardo G, Fernandez-Chacon R, Schluter OM, Sudhof TC (2005) Alpha-synuclein cooperates with CSPalpha in preventing neurodegeneration. Cell 123: 383–396. 16269331 10.1016/j.cell.2005.09.028

[52] Rickman C, Davletov B (2005) Arachidonic acid allows SNARE complex formation in the presence of Munc18. Chem Biol 12: 545–553. 15911375 10.1016/j.chembiol.2005.03.004

[53] Thayanidhi N, Helm JR, Nycz DC, Bentley M, Liang Y, et al (2010) Alpha-synuclein delays endoplasmic reticulum (ER)-to-Golgi transport in mammalian cells by antagonizing ER/Golgi SNAREs. Mol Biol Cell 21: 1850–1863. 20392839 10.1091/mbc.E09-09-0801 PMC2877643

[54] Burgalossi A, Jung S, Meyer G, Jockusch WJ, Jahn O, et al (2010) SNARE Protein Recycling by ±SNAP and ^2^SNAP Supports Synaptic Vesicle Priming. Neuron 68: 473–487. 21040848 10.1016/j.neuron.2010.09.019

[55] Anderson JP, Walker DE, Goldstein JM, de Laat R, Banducci K, et al (2006) Phosphorylation of Ser-129 is the dominant pathological modification of alpha-synuclein in familial and sporadic Lewy body disease. J Biol Chem 281: 29739–29752. 16847063 10.1074/jbc.M600933200

[56] Farrer M, Maraganore DM, Lockhart P, Singleton A, Lesnick TG, et al (2001) alpha-Synuclein gene haplotypes are associated with Parkinson's disease. Hum Mol Genet 10: 1847–1851. 11532993 10.1093/hmg/10.17.1847

[57] Zarranz JJ, Alegre J, Gomez-Esteban JC, Lezcano E, Ros R, et al (2004) The new mutation, E46K, of alpha-synuclein causes Parkinson and Lewy body dementia. Ann Neurol 55: 164–173. 14755719 10.1002/ana.10795

[58] Liu J, Cavalli LR, Haddad BR, Papadopoulos V (2003) Molecular cloning, genomic organization, chromosomal mapping and subcellular localization of mouse PAP7: a PBR and PKA-RIalpha associated protein. Gene 308: 1–10. 12711385 10.1016/s0378-1119(03)00453-0

[59] Frank-Cannon TC, Tran T, Ruhn KA, Martinez TN, Hong J, et al (2008) Parkin deficiency increases vulnerability to inflammation-related nigral degeneration. J Neurosci 28: 10825–10834. 18945890 10.1523/JNEUROSCI.3001-08.2008 PMC2603252

[60] Shapiro SS, WIlk MB (1965) An analysis of varianace test for normality. Biometrikal 52: 591–611.

[61] Levene H, Ingram O, Hotelling H, et al. (1960) Contributions to Probability and Statistics: Essays in Honor of Harold Htelling.: Standford Univeristy Press.

